# Heterogeneous Associations Between Frequent Virtual Communication and Loneliness Among Older Adults: Observational Analysis

**DOI:** 10.2196/96187

**Published:** 2026-08-07

**Authors:** Daisuke Kato, Ichiro Kawachi, Katsunori Kondo, Atsushi Nakagomi, Koichiro Shiba

**Affiliations:** 1 Takemi Program in International Health Harvard T.H. Chan School of Public Health Boston, MA United States; 2 Center for Preventive Medical Sciences Chiba University Chiba, Chiba Japan; 3 Section of Global Health and Preventive Medicine Institute of Global Well-being Science Hirosaki University Hirosaki, Aomori Japan; 4 Department of Social Medicine and Behavioral Science Graduate School of Medicine, Division of Health Sciences The University of Osaka Suita, Osaka Japan; 5 Department of Social and Behavioral Sciences Harvard T.H. Chan School of Public Health Boston, MA United States; 6 Research Department Institute for Health Economics and Policy Minato-ku, Tokyo Japan; 7 Department of Epidemiology Boston University School of Public Health Boston, MA United States

**Keywords:** loneliness, human-computer interaction, older adults, observational study, machine learning, heterogeneity

## Abstract

**Background:**

Loneliness is a long-standing and widely acknowledged public health problem among older adults, yet evidence remains mixed regarding how virtual communication is associated with loneliness. Less is known about whether this association varies across baseline profiles among older adults.

**Objective:**

This study aimed to examine the association between frequent virtual communication with family, friends, or acquaintances and loneliness among older adults in Japan and explore whether this association varied across baseline profiles.

**Methods:**

Using linked 2019 and 2022 data from the Japan Gerontological Evaluation Study, we conducted an observational analysis of community-dwelling Japanese adults aged 65 years or older (N=4807). Baseline covariates were measured in 2019. Frequent virtual communication was assessed in 2022 based on retrospective reports of internet or email use for communication with family, friends, or acquaintances in the preceding year; frequent use was defined as use at least 2 to 3 times per week. Participants not meeting this definition were categorized as nonusers or infrequent users. Loneliness was measured in 2022 using the 3-item UCLA Loneliness Scale (range 3-9). We used targeted maximum likelihood estimation with *SuperLearner* and 42 baseline covariates to estimate the sample average–adjusted association and a generalized random forest approach to explore heterogeneity in the estimated association across baseline profiles. Supplementary analyses examined baseline loneliness adjustment in an alternative linked sample, a dichotomized loneliness outcome, propensity score overlap, and model calibration.

**Results:**

The analytic sample included 4807 participants, of whom 1616 (33.6%) met the frequent virtual communication definition. After adjusting for observed baseline covariates, frequent virtual communication was associated with a difference of −0.136 in loneliness scores (95% CI −0.194 to −0.078). Generalized random forest analyses suggested exploratory heterogeneity in the estimated association across baseline profiles; most individual-level forest predictions for the estimated association were below 0 (4519/4807, 94.0%). In supplementary analyses, the association remained in the same direction after adjustment for baseline loneliness in an alternative linked sample (adjusted mean difference −0.117, 95% CI −0.173 to −0.061) and when loneliness was dichotomized (odds ratio for loneliness 0.734, 95% CI 0.597-0.904). Propensity score diagnostics showed overlap between exposure groups, although overlap was limited in the tails.

**Conclusions:**

In this observational analysis of older adults in Japan, frequent virtual communication was associated with modestly lower loneliness, and generalized random forest analyses suggested exploratory heterogeneity across baseline profiles. The findings should be interpreted cautiously because the exposure was retrospectively reported, loneliness was measured in the same survey wave, baseline loneliness was not available in the main analytic sample, and sample selection may have affected generalizability. The findings should not be interpreted as evidence of causal effects.

## Introduction

Loneliness—a distressing experience that arises from a perceived discrepancy between one’s desired and actual level of social connectedness—has emerged as an urgent public health concern [1-3].

Loneliness is common among older adults; recent global estimates suggest that approximately 1 in 10 older people experience loneliness [3], and prior epidemiological work has identified this demographic as a high-risk population because social isolation and mobility limitations can exacerbate loneliness [4]. Japan provides an important context for examining virtual communication and loneliness because it has one of the most rapidly increasing aging populations, and loneliness and digital exclusion in later life are major public health concerns. The Japan Gerontological Evaluation Study (JAGES) data include rich information on social participation, social capital, health, and digital engagement, allowing us to examine this association among community-dwelling older adults. Loneliness has been linked to a range of adverse health outcomes, including depression, anxiety, cognitive decline, and even increased mortality risk [5-7].

Virtual platforms such as video calls, social media, and online support may help older adults maintain social connections when in-person interaction is constrained. The COVID-19 pandemic and resulting social distancing policies highlighted the potential role of digital platforms in maintaining social connections when face-to-face interactions are restricted [8]. Virtual communication may be related to loneliness through several plausible pathways, including helping older adults maintain emotionally meaningful contact, broadening access to geographically distant social ties, and supporting routine interaction among individuals with mobility limitations. These mechanisms are plausible but were not directly tested in the present study. Digital communication tools are increasingly used by older adults, propelled by wider internet access and improved digital literacy [9,10], although adoption remains uneven because of differences in digital access, skills, and social resources [9,10]. However, previous studies have reported mixed evidence on how technology-based social interactions are associated with loneliness among older adults: some have reported lower loneliness or improved social connectedness [11], whereas others have found little or no consistent improvement in loneliness [12]. One possible explanation for these mixed findings is that the association between virtual communication and loneliness may vary across baseline social, demographic, health, and neighborhood profiles [13]. Previous studies have often focused on estimating average associations and have treated older adults as a relatively homogeneous population. Less is known about whether the association between virtual communication and loneliness differs across baseline social, demographic, health, and neighborhood profiles. Because the survey assessed virtual communication using frequency categories, this study focused on a frequency-based operationalization of regular virtual communication. This approach allowed us to quantitatively assess differential degrees of virtual communication without substantially increasing respondent burden.

In this study, we examined the association between frequent virtual communication and loneliness among older adults in Japan and explored whether this association varied across baseline profiles using linked 2019 and 2022 JAGES data. This study focused specifically on internet-mediated communication rather than interpersonal communication in general. Because the exposure was retrospectively reported, loneliness was assessed in the same survey wave, and the exposure does not correspond to a precisely specified intervention, we interpreted the analyses as adjusted associations rather than causal effects. We used a generalized random forest approach to explore whether the estimated association between frequent virtual communication and loneliness varied across baseline demographic, health, social, and neighborhood profiles.

## Methods

### Study Design and Sample

This study used a 2-wave observational design using linked 2019 and 2022 JAGES data. The JAGES survey was conducted as a mailed questionnaire survey rather than an online survey. Therefore, participation was not restricted to internet users. Eligible participants were community-dwelling older adults aged 65 years or older who responded to the 2019 JAGES survey, were followed up on in 2022, and received the internet use questionnaire used to define frequent virtual communication. Baseline covariates were measured in the 2019 JAGES survey. Frequent virtual communication was assessed in the 2022 survey based on retrospective reports of internet or email use for communication in the preceding year. Loneliness was assessed in the same 2022 survey wave. Therefore, although baseline covariates preceded the exposure assessment, the temporal ordering between the retrospectively reported exposure window and loneliness assessment cannot be fully established. The assumed temporal and reporting structure is illustrated in Figure S1 in Multimedia Appendix 1. The JAGES is an ongoing, population-based cohort study of community-dwelling Japanese adults aged 65 years or older initiated in 2010, with mail surveys conducted approximately every 3 years. In 2019, questionnaires were mailed to approximately 387,000 residents across 66 municipalities, and approximately 266,000 responses were returned (response rate=68.7%). In the 2022 follow-up wave, approximately 340,000 questionnaires were mailed across 76 municipalities, and approximately 229,000 responses were returned (response rate=67.3%).

**Figure 1:**
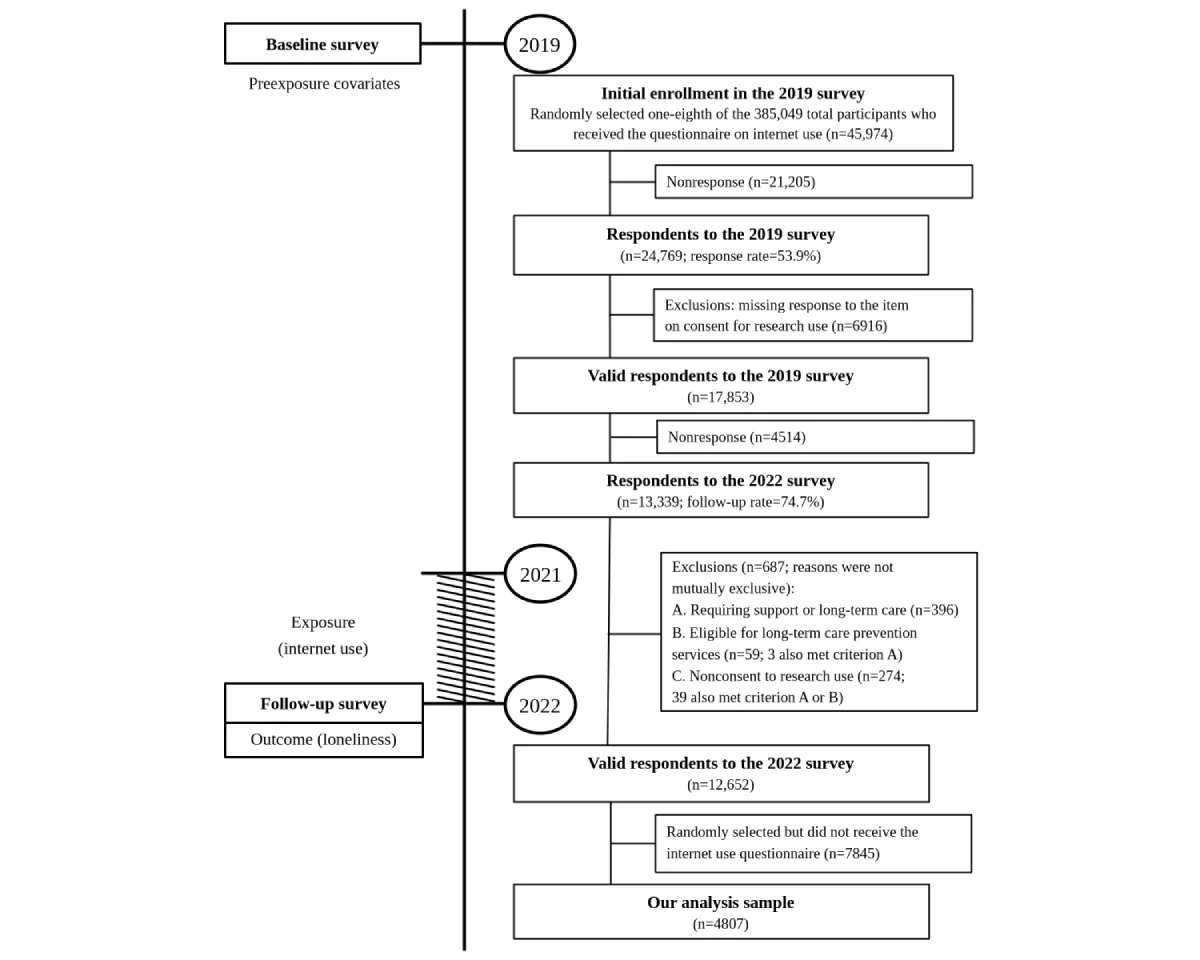
Flow diagram of the analytic sample from the 2019 and 2022 waves of the Japan Gerontological Evaluation Study. This study examined the association between frequent virtual communication and loneliness among community-dwelling older adults in Japan. The diagram reports the available counts for nonresponse, consent-related exclusions, 2022 exclusion reasons, respondents who did not receive the 2022 internet use questionnaire, and the final analytic sample. Exclusion reasons in 2022 were not mutually exclusive.

The internet use module was distributed to a random subsample of 45,974 participants in the 2019 survey. Of these 45,974 participants, 21,205 (46.1%) did not respond, and 24,769 (53.9%) responded. Of the 24,769 participants who responded, we excluded 6916 (27.9%) with missing responses to the item on consent for research use, leaving 17,853 (72.1%) valid respondents in 2019. In 2022, a total of 4514 participants did not respond, and 13,339 responded. Of the 13,339 participants who responded, we excluded 687 (5.2%); the exclusion reasons were not mutually exclusive and included requiring support or long-term care (n=396, 57.6%), eligibility for long-term care prevention services (n=59, 8.6%; 3/59, 5.1% also met the first criterion), and nonconsent to research use (n=274, 39.9%; 39/274, 14.2% also met the first or second criterion). Of the 12,652 valid 2022 respondents, 7845 (62.0%) were randomly selected but did not receive the 2022 internet use questionnaire, leaving a final analytic sample of 4807 (38.0%). The sample selection flow is shown in Figure 1.

### Ethical Considerations

Ethics approval was obtained from the ethics committee of Chiba University School of Medicine (2493), the National Center for Geriatrics and Gerontology (992), and Nihon Fukushi University (10-05). Written informed consent was assumed with the voluntary return of the questionnaire. Respondents who indicated that they did not agree to participate in the study were excluded from the analysis.

### Measurement

The analytical framework consisted of baseline covariates measured in 2019, frequent virtual communication retrospectively assessed in 2022 for the preceding year, and loneliness assessed in 2022. The 2019 variables were treated as baseline covariates, frequent virtual communication was the exposure variable, and loneliness was the outcome.

#### Exposure: Frequent Virtual Communication

In the 2022 wave, participants were asked how often they had used the internet or email in the preceding year. We defined frequent use as use at least 2 to 3 times per week. This threshold was based on the response categories available in the questionnaire and was selected as a pragmatic indicator of frequent rather than occasional use. A stricter threshold would have defined a different exposure contrast and excluded participants who reported using the internet or email 2 to 3 times per week. Among those reporting internet or email use, participants were also asked whether their purposes of use included communication with family, friends, or acquaintances. The questionnaire listed examples, such as email, Line, Zoom, and video calls, but the item did not separately identify each platform or communication modality. Frequent virtual communication was not measured using a stand-alone validated scale; it was operationalized using these questionnaire items on frequency and communication purpose. We therefore defined frequent virtual communication as using the internet or email at least 2 to 3 times per week and reporting communication with family, friends, or acquaintances as one purpose of use. Participants who did not meet this definition were classified as nonusers or infrequent users. The reference group included participants who did not use internet or email; those who used the internet or email less frequently than the frequent use threshold; and those who used internet or email at least 2 to 3 times per week but did not report communication with family, friends, or acquaintances as a purpose of use. The exposure was defined as internet-mediated communication and did not include telephone contact or face-to-face interaction. The survey item did not identify the primary reason for communication or distinguish among emotional support, routine coordination, and information sharing.

#### Outcome: Loneliness

We measured loneliness as of the 2022 survey using the 3-item UCLA Loneliness Scale [14,15]. Each item (eg, “How often do you feel left out?”) was rated on a 3-point Likert scale. Scores range from 3 (lowest loneliness) to 9 (highest loneliness). We treated loneliness as a continuous variable following a previous study on loneliness using JAGES data [16]. Internal consistency in our analytic sample was high (Cronbach α=0.912 [ordinal based on polychoric correlations], 95% bootstrap CI 0.904-0.920).

#### Covariates

We used measures available in 2019 as covariates and selected 42 potential ones covering demographic, socioeconomic, health, psychosocial, and behavioral domains. These variables were selected based on prior studies suggesting that both internet use and loneliness may be influenced by a wide range of individual characteristics [17,18]. We also assessed civic participation, reciprocity, and social cohesion using participants’ individual-level responses to the JAGES health-related social capital item, for which prior JAGES work has reported evidence supporting reliability and validity among older Japanese adults [19]. Civic participation is the summed frequency of involvement in volunteer, sports, hobby, cultural, and skill-sharing groups. Social cohesion captures neighborhood trust, shared norms of mutual aid, and attachment to the area. Reciprocity indexes the giving and receiving of emotional and instrumental support, reflecting mutual assistance within the community. Higher scores indicate greater perceived neighborhood social capital. In the present study, these established indicators were used as baseline covariates to capture perceived neighborhood social capital rather than constructing new scales. We adjusted for general internet use in 2019 as a proxy for baseline digital engagement [20]. This item did not specifically measure prior virtual communication. In the 2019 JAGES survey, all respondents received the core items, and 1 of 8 additional questionnaire modules was randomly assigned to each respondent to reduce respondent burden. Therefore, module-specific items were by design available only for respondents assigned to the corresponding module. Because the general internet use item and the loneliness items were included in different 2019 survey submodules, baseline loneliness was not measured in the main analytic sample, which required the general internet use item.

To assess robustness to baseline loneliness adjustment, we constructed an alternative linked sample by starting from 2019 respondents with baseline loneliness data; linking them to 2022 follow-up data; and applying the corresponding 2022 eligibility, exposure, and outcome availability criteria. This yielded 4682 participants. Because the general internet use covariate was unavailable in this alternative sample by design, this analysis was interpreted as a supplementary robustness check rather than a direct replacement for the main analysis. A full list of variables is provided in Table S1 in Multimedia Appendix 1.

### Statistical Analysis

First, we used targeted maximum likelihood estimation (TMLE) via the *SuperLearner* function and 42 baseline covariates to estimate the sample average–adjusted association between frequent virtual communication and loneliness [21-23]. Because frequent virtual communication was retrospectively reported in the 2022 survey and loneliness was measured in the same survey wave, we interpreted the estimates as observational associations rather than definitive causal effects. TMLE combines an outcome model and an exposure model and has a doubly robust structure. This means that the estimator can remain consistent if either the outcome model or the exposure model is correctly specified under standard assumptions. For example, the estimator may remain consistent if the outcome model for loneliness is misspecified but the exposure model for frequent virtual communication is correctly specified, or vice versa. However, TMLE does not address unmeasured confounding, recall bias, or ambiguity in temporal ordering. The *SuperLearner* library included generalized linear models, neural networks, and gradient boosting [24-26]. This learner set was selected pragmatically to balance interpretability, flexibility for nonlinear patterns, ability to capture higher-order interactions, and computational feasibility. We do not claim that this library is uniquely optimal. Generalized linear models were included to provide a parsimonious main-effect structure, neural networks were included to allow for flexible nonlinear patterns, and gradient boosting was included to capture nonlinearities and higher-order interactions. We used the *ltmle* and *SuperLearner* packages in R (R Foundation for Statistical Computing) [21,23].

Next, we used a generalized random forest approach to explore heterogeneity in the estimated association across baseline profiles. Generalized random forests were selected for the heterogeneity analysis because they can flexibly explore variation in adjusted associations across many covariates and potential interactions while reducing dependence on any single tree-based partition. Compared with a single regression tree or other simpler tree-based subgroup approaches, this approach averages over many trees, uses honesty procedures to reduce overfitting, provides individual-level forest predictions for ranking participants, and enables calibration diagnostics for assessing heterogeneity in the estimated associations [27,28]. We acknowledge that other estimators for heterogeneous associations exist [29], but assessing the comparative performance across different algorithms is beyond the scope of this study.

Conditional average treatment effect (CATE) denotes the conditional average treatment effect estimand used by the generalized random forest algorithm; estimated CATEs denote individual-level forest predictions used to rank participants. Because this was an observational association analysis, the estimated CATEs are interpreted as exploratory heterogeneity in adjusted associations, not as definitive causal effects. In growing trees, the algorithm randomly splits a subsample into 2 halves, uses the first half to determine the partitioning, and uses the second half to estimate predictions within each leaf; this process reduces overfitting in tree predictions. We implemented the generalized random forest using the R package *grf* [30]. We used 10-fold cross-fitting so that predictions for each fold were obtained from trees trained on data excluding observations from that fold. Before growing 10,000 trees, we used out-of-bag predictions and selected tuning parameters via cross-validation, including the subsampling fraction, number of candidate variables at each split, target minimum number of observations in each leaf, fraction of data used for determining splits, pruning settings for empty leaves, and split imbalance parameters.

Model calibration was assessed using the test_calibration() function in the *grf* package. This best linear predictor analysis evaluated calibration of the average forest prediction using the mean forest coefficient and evidence of heterogeneity in the estimated CATEs using the differential forest coefficient. We also used quartiles to summarize estimated CATE profiles for interpretability. To construct the quartile summaries, participants were ranked within each fold according to their estimated CATEs and divided into quartiles (quartile 1 to quartile 4). These quartile summaries were used descriptively and were not intended as the formal calibration assessment.

Finally, we conducted several supplementary analyses and diagnostics. First, we used an alternative linked sample with baseline loneliness to examine whether additional adjustment for baseline loneliness changed the estimated association. Second, as a supplementary sensitivity analysis, we repeated the analysis using a dichotomized loneliness outcome. Following prior studies using the 3-item UCLA Loneliness Scale [31,32], scores of 6 to 9 were classified as lonely, and scores of 3 to 5 were classified as not lonely. This dichotomization was used only for a supplementary sensitivity analysis, and the continuous loneliness score remained the primary outcome. Third, we evaluated propensity score overlap between frequent users and nonusers or infrequent users. Fourth, as described above, we assessed calibration of the generalized random forest estimates using the test_calibration() function in the *grf* package.

We imputed missing values for all analysis variables using *missForest*, a random forest–based machine learning method [33]. We used *missForest* because the analysis variables included mixed variable types, including continuous, binary, ordinal, and categorical variables. *missForest* can accommodate nonlinear relationships and interactions among mixed-type variables without requiring separate parametric models for each variable type. The final analytical models were fitted using the imputed analytic dataset. The missing categories shown in Table S2 in Multimedia Appendix 1 refer to preimputation missingness in the original data columns and were presented for descriptive purposes only; missingness was not treated as a separate category in the final analytical models. The overall proportion of missing data was relatively small (<10%), as shown in Table S2 in Multimedia Appendix 1. All analyses were conducted in R (version 3.6.0) using the *ltmle*, *SuperLearner*, *missForest*, and *grf* packages.

## Results

Selected baseline characteristics of the analytic sample and loneliness in 2022 are shown in Table 1, and full descriptive statistics are provided in Table S2 in Multimedia Appendix 1. The analytic sample included 4807 participants, of whom 1616 (33.6%) met the frequent virtual communication definition. Compared with nonusers or infrequent users, frequent virtual communication users tended to be younger, had higher educational attainment and household income, reported fewer depressive symptoms, and more often had frequent contact with friends. Of the 3191 participants in the group of nonusers or infrequent users, 1962 (61.5%) did not use the internet or email; 670 (21%) used the internet or email less frequently than the frequent use threshold; and 559 (17.5%) used the internet or email at least 2 to 3 times per week but did not report communication with family, friends, or acquaintances as a purpose of use.

**Table 1:** Baseline covariates measured in 2019 and loneliness assessed in 2022 by frequent virtual communication status among community-dwelling older adults in Japan in the imputed analytic sample (N=4807).

Characteristic	Total	Frequent virtual communication users^a^ (n=1616)	Nonusers or infrequent users (n=3191)
Loneliness in 2022 (3-9)^b^, mean (SD)	4.2 (1.4)	3.9 (1.2)	4.3 (1.5)
Age (y), mean (SD)	73.7 (6)	71.2 (4.9)	74.9 (6.1)
Gender (women), n (%)	2548 (53)	926 (57.3)	1622 (50.8)
Marital status (married), n (%)	3607 (75)	1303 (80.6)	2304 (72.2)
Educational attainment (≥13 y), n (%)	1360 (28.3)	686 (42.5)	674 (21.1)
Annual household income (¥10,000; US $1=¥164 as of July 27, 2026), mean (SD)	242 (146)	287 (153)	220 (137)
Alcohol consumption (yes), n (%)	1922 (40)	710 (43.9)	1212 (38)
Smoking status (yes), n (%)	452 (9.4)	129 (8)	323 (10.1)
Number of natural teeth (≥20), n (%)	2544 (52.9)	1105 (68.4)	1439 (45.1)
Frequency of going out (once a week or more), n (%)	4679 (97.3)	1602 (99.1)	3077 (96.4)
Civic participation (0-5)^c^, mean (SD)	1.1 (1.2)	1.4 (1.3)	0.9 (1.2)
Reciprocity (0-3)^c^, mean (SD)	2.7 (0.6)	2.8 (0.5)	2.7 (0.7)
Social cohesion (0-3)^c^, mean (SD)	2.1 (1.1)	2.2 (1)	2 (1.1)
Living alone, n (%)	571 (11.9)	161 (10)	410 (12.8)
Self-rated health (very good), n (%)	728 (15.1)	296 (18.3)	432 (13.5)
GDS-15^d^ (0-15), mean (SD)	2.9 (3.0)	2.2 (2.5)	3.3 (3.1)
Frequency of meeting friends (≥4 times weekly), n (%)	725 (15.1)	305 (18.9)	420 (13.2)
Internet use in 2018-2019 (a few times a month or less), n (%)	2635 (54.8)	282 (17.5)	2353 (73.7)

^a^Frequent virtual communication was defined using a frequency item for internet or email use and a communication purpose item for communication with family, friends, or acquaintances at least 2 to 3 times per week in the preceding year. The communication purpose item included examples such as email, Line, Zoom, and video calls but did not separately identify each platform or modality. Nonusers or infrequent users were defined as participants who did not meet this frequent virtual communication definition.

^b^Loneliness was assessed in 2022 using the 3-item UCLA Loneliness Scale (range 3-9), with higher scores indicating greater loneliness.

^c^The 3 key dimensions of social capital.

^d^GDS-15: 15-item Geriatric Depression Scale.

**Figure 2:**
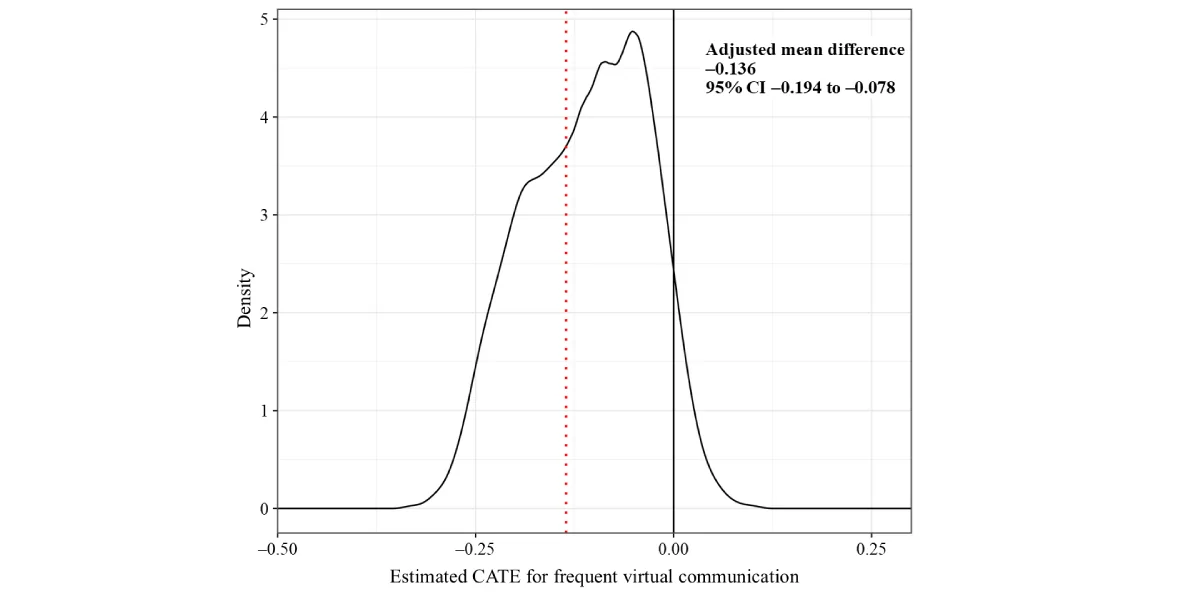
Distribution of estimated conditional average treatment effects (CATEs) for the association between frequent virtual communication and loneliness among older adults in Japan.

**Figure 3:**
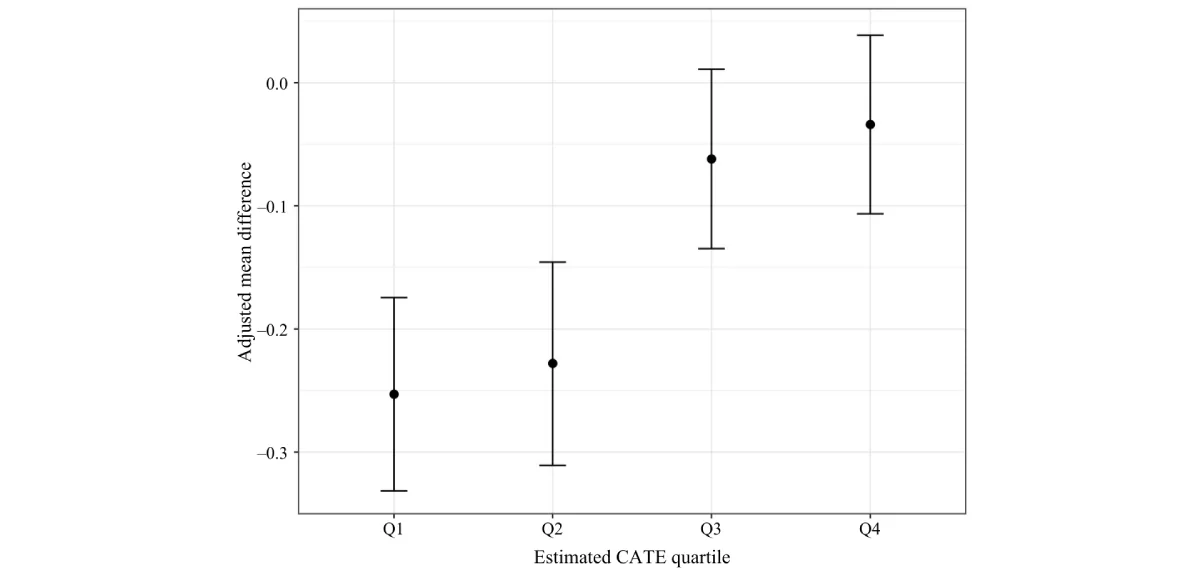
Generalized random forest quartile (Q) summary for estimated conditional average treatment effects (CATEs) for the association between frequent virtual communication and loneliness among older adults in Japan.

After adjusting for observed baseline covariates, frequent virtual communication was associated with a difference of −0.136 in loneliness scores (95% CI −0.194 to −0.078). Generalized random forest analyses suggested exploratory heterogeneity in the estimated association across baseline profiles; most individual-level forest predictions for the estimated association were below 0 (4519/4807, 94.0%). The distribution of the estimated CATEs is shown in Figure 2. Generalized random forest calibration was assessed using the test_calibration() function in the *grf* package. The coefficient for the mean forest prediction was 1.067 (*P*=.002), suggesting calibration of the average forest prediction. The coefficient for the differential forest prediction was 1.361 (*P*<.001), providing evidence of heterogeneity in the estimated CATEs. The adjusted mean differences by quartile of the estimated CATEs are shown in Figure 3. Selected baseline characteristics across the quartiles of the estimated CATEs are shown in Table 2; the full list of baseline characteristics is provided in Table S3 in Multimedia Appendix 1. Supplementary analyses are shown in Tables S4-S6 and Figure S2 in Multimedia Appendix 1. In the supplementary analysis using the alternative linked sample with baseline loneliness, frequent virtual communication remained associated with lower loneliness after additional adjustment for baseline loneliness (adjusted mean difference −0.117, 95% CI −0.173 to −0.061; Table S4 in Multimedia Appendix 1). In the dichotomized-outcome sensitivity analysis, frequent virtual communication was associated with lower odds of loneliness (odds ratio 0.734, 95% CI 0.597-0.904; Table S5 in Multimedia Appendix 1). Propensity score overlap diagnostics are shown in Table S6 and Figure S2 in Multimedia Appendix 1. The distributions overlapped across exposure groups, although overlap was limited in the tails. Variable importance values from the generalized random forest are presented in Table S7, and communication modality-related variables available in the 2022 questionnaire are summarized in Table S8, both in Multimedia Appendix 1.

**Table 2:** Selected baseline covariates measured in 2019 and loneliness assessed in 2022 stratified by quartiles of estimated conditional average treatment effects (CATEs) for the association between frequent virtual communicationa and loneliness among older adults in Japanb.

Baseline characteristic	Quartile 1 (n=1207)	Quartile 2 (n=1200)	Quartile 3 (n=1200)	Quartile 4 (n=1200)
Estimated CATEs, mean (SD)	−0.21 (0.03)	−0.13 (0.02)	−0.07 (0.02)	−0.02 (0.03)
Loneliness in 2022 (3-9)^c^, mean (SD)	4.5 (1.6)	4.3 (1.5)	4.1 (1.4)	3.9 (1.3)
Age (y), mean (SD)	73.2 (5.9)	74.1 (6.1)	74.0 (6.0)	73.4 (5.8)
Gender (women), n (%)	51 (4)	376 (31)	960 (80)	1161 (97)
Marital status (married), n (%)	989 (82)	918 (76)	830 (69)	870 (72)
Educational attainment (≥13 y), n (%)	515 (43)	308 (26)	298 (25)	239 (20)
Annual household income (¥10,000; US $1= ¥164 as of July 27, 2026), mean (SD)	240 (154)	227 (143)	233 (135)	269 (150)
Alcohol consumption (yes), n (%)	266 (22)	423 (35)	737 (61)	828 (69)
Smoking status (yes), n (%)	164 (14)	200 (17)	77 (6)	11 (1)
Number of natural teeth (≥20), n (%)	594 (49)	563 (47)	615 (51)	772 (64)
Frequency of going out (once a week or more), n (%)	1167 (97)	1163 (97)	1172 (98)	1177 (98)
Civic participation^d^ (0-5), mean (SD)	0.8 (1.1)	1.0 (1.2)	1.2 (1.3)	1.3 (1.3)
Reciprocity^d^ (0-3), mean (SD)	2.5 (0.8)	2.7 (0.6)	2.7 (0.5)	2.9 (0.3)
Social cohesion^d^ (0-3), mean (SD)	2.0 (1.1)	2.1 (1.1)	2.1 (1.1)	2.1 (1.0)
Living alone (yes), n (%)	128 (11)	139 (12)	167 (14)	137 (11)
Self-rated health (very good), n (%)	162 (13)	221 (18)	193 (16)	152 (13)
GDS-15^e^ (0-15), mean (SD)	3.2 (3.3)	3.1 (3.2)	2.8 (2.8)	2.7 (2.5)
Frequency of meeting friends (≥4 times weekly), n (%)	164 (14)	172 (14)	229 (19)	160 (13)
Internet use in 2018-2019 (a few times a month or less), n (%)	636 (53)	688 (57)	701 (58)	610 (51)

^a^Frequent virtual communication was defined using a frequency item for internet or email use and a communication purpose item for communication with family, friends, or acquaintances at least 2 to 3 times per week in the preceding year. The communication purpose item included examples such as email, Line, Zoom, and video calls but did not separately identify each platform or modality. Nonusers or infrequent users were defined as participants who did not meet this frequent virtual communication definition.

^b^Quartile 1 to quartile 4 represent quartiles of the estimated CATEs. Quartile 1 had the most negative estimated CATEs, whereas quartile 4 had estimated CATEs closest to the null. CATE denotes the conditional average treatment effect estimand; estimated CATEs denote the individual-level forest predictions used for stratification. Because this study was an observational association analysis, the estimated CATEs are interpreted as exploratory heterogeneity in adjusted associations, not as definitive causal effects.

^c^Loneliness was assessed in 2022 using the 3-item UCLA Loneliness Scale (range 3-9), with higher scores indicating greater loneliness.

^d^The 3 key dimensions of social capital.

^e^GDS-15: 15-item Geriatric Depression Scale.

The quartile 1 group, which had the most negative estimated CATEs (mean estimated CATE −0.21, SD 0.03), had a higher proportion of men compared to quartile 4 (51/1207, 4% vs 1161/1200, 97%) and higher educational attainment (515/1207, 43% of participants had ≥13 years of education vs 239/1200, 20%). Compared to quartile 4, the quartile 1 group also had a lower prevalence of alcohol consumption (266/1207, 22% vs 828/1200, 69%), a higher prevalence of current smoking (164/1207, 14% vs 11/1200, 1%), and lower levels of civic participation (mean 0.8, SD 1.1 vs mean 1.3, SD 1.3) and reciprocity (mean 2.5, SD 0.8 vs mean 2.9, SD 0.3) in their own neighborhoods, whereas they had comparable social cohesion (mean 2.0, SD 1.1 vs mean 2.1, SD 1.0). The quartile 1 group also reported greater baseline depressive symptoms (15-item Geriatric Depression Scale: mean 3.2, SD 3.3 vs mean 2.7, SD 2.5). We characterized baseline profiles across quartiles of the estimated CATEs. These comparisons were used descriptively to summarize profiles for which frequent virtual communication was estimated to be more or less strongly associated with loneliness. They should not be interpreted as evidence that any single characteristic independently modified the association.

## Discussion

### Principal Findings

In this observational analysis of community-dwelling older adults in Japan, frequent virtual communication was associated with modestly lower loneliness. Generalized random forest analyses suggested exploratory heterogeneity in the estimated association across baseline profiles. The quartile profiles indicated that participants with the most negative estimated CATEs differed in several baseline characteristics from those with estimated CATEs closer to the null, but these descriptive comparisons should not be interpreted as evidence that any single characteristic independently modified the association.

Our finding that frequent virtual communication was associated with lower loneliness complements those of previous studies on information and communications technology interventions and social connectedness [11]. Virtual communication may be related to loneliness through several plausible pathways. It may help older adults maintain emotionally meaningful contact when in-person interaction is constrained, broaden access to geographically distant social ties, and support routine interaction among individuals with mobility limitations. Our findings can also be interpreted in relation to networked individualism and social capital perspectives [34]. Virtual communication may allow older adults to maintain social ties beyond geographically proximate networks and may supplement locally available social resources. However, these interpretations remain speculative because the present study did not directly examine communication content, tie strength, or mechanisms linking virtual communication to loneliness.

Previous studies have reported mixed evidence on information and communications technology interventions and loneliness [11]. The exploratory heterogeneity observed in this study may partly help explain why average associations differ across studies, but the quartile profiles should not be interpreted as identifying independent effect modifiers. Profiles with more negative estimated CATEs included participants with several baseline characteristics, such as a higher proportion of men, higher educational attainment, greater depressive symptoms, lower civic participation and reciprocity, and a higher prevalence of current smoking. These patterns are descriptive and hypothesis generating.

The adjusted average association (−0.136, 95% CI −0.194 to −0.078) was small in magnitude. Therefore, it should not be interpreted as evidence of a large change in loneliness at the individual level. However, because internet-mediated communication is scalable, even modest population-level associations may be relevant from a public health perspective [35]. This interpretation remains cautious because the present study was observational. A frequency-based measure is useful in large-scale epidemiological surveys because it allows virtual communication use to be quantified without substantially increasing respondent burden. However, frequency alone does not capture the quality, duration, emotional tone, reciprocity, or relational context of communication. Therefore, frequent virtual communication should not be interpreted as equivalent to emotionally meaningful or supportive communication. Virtual communication may also involve risks for older adults, including exposure to fraud, misinformation, privacy concerns, and the burden of navigating digital tools [36]. Any practical application of digital communication should therefore consider digital literacy, safety, accessibility, and support for older adults. Future studies should distinguish communication modalities such as text-based messaging, email, video calls, and telephone contact. They should also measure communication quality, emotional tone, tie strength, and relational context. More prospective designs with baseline loneliness and repeated assessments of communication and loneliness are needed to clarify temporal ordering. Replication in other countries and populations is also warranted. However, the present exposure definition relied on JAGES-specific survey items on frequency and purpose of internet or email use. Equivalent measures may not be available in other datasets, and harmonization across datasets would require separate validation work.

### Limitations

Several limitations merit consideration. First, the exposure was retrospectively reported in the 2022 survey for the preceding year, whereas loneliness was assessed in the same survey wave. Therefore, true temporal precedence cannot be established. Current loneliness may have influenced participants’ recall of prior virtual communication, and this potential recall bias should be considered when interpreting the findings. Second, baseline loneliness was not available in the main analytic sample, and residual confounding or reverse causation due to unmeasured baseline loneliness remains possible. Although adjustment for baseline general internet use may partly capture prior digital engagement that could be related to both baseline loneliness and later virtual communication, this item did not specifically measure prior virtual communication and, therefore, does not eliminate this concern. The supplementary analysis using an alternative linked sample with baseline loneliness showed an association in the same direction. However, while assignment of the 2019 questionnaire modules was at random, patterns of nonresponse to the baseline loneliness module might differ from patterns of response to the submodule used in the main analysis, leading to distinct selection bias. Third, participant characteristics may have changed between the 2019 baseline survey and the 2021 to 2022 exposure window. Such changes, including changes in health, social relationships, or digital access, may have introduced time-varying confounding that was not captured by baseline covariates. Fourth, our primary exposure combined heterogeneous communication modes and did not distinguish asynchronous text-based communication from real-time video interaction. This aggregation may have diluted or obscured modality-specific associations. The survey item also did not identify the primary reason for communication or distinguish communication content, such as emotional support, routine coordination, and information sharing. Therefore, we could not evaluate whether the association differed by communication purpose or content. In addition, general internet use in 2019 was used as a proxy for baseline digital engagement and did not specifically measure prior virtual communication. Fifth, the reference group was heterogeneous and included true nonusers; infrequent internet or email users; and participants who used the internet or email frequently but did not report communication with family, friends, or acquaintances as a purpose of use. Therefore, the comparison was not between frequent virtual communication and exclusive nonuse. This heterogeneity may have diluted the estimated association. Sixth, because the exposure did not include telephone contact or face-to-face interaction, the findings should be interpreted as specific to internet-mediated communication and should not be generalized to interpersonal communication or remote communication more broadly. Seventh, the analytic sample (N=4807) was drawn from the 2019 survey respondents available in the distributed individual-level data (n=24,769); baseline covariates were unavailable for the preresponse initial enrollment group (n=45,974). Compared with the 2019 respondent sample, the analytic sample differed in several baseline characteristics (Table S9 in Multimedia Appendix 1). This attrition may limit generalizability and may have affected the sample average association in either direction depending on whether underrepresented participants would have shown weaker or stronger associations with frequent virtual communication. Attrition may also have affected the estimated heterogeneity patterns. Although we evaluated propensity score distributions and observed reasonable overlap except in the tails, residual extrapolation may remain in some covariate regions. Eighth, the heterogeneity analysis may be affected by correlated covariates and noisy predictors, and the estimated CATE profiles should therefore be interpreted as exploratory. Variable importance values from the generalized random forest reflect how often variables contributed to splitting within the forest algorithm. High variable importance does not necessarily indicate that a variable is a conceptually important or independent source of heterogeneity. We did not compare estimates across multiple heterogeneity estimators, and such comparisons should be considered in future work.

### Conclusions

Frequent virtual communication was associated with modestly lower loneliness among older adults in Japan. The association varied across baseline profiles, but these heterogeneity findings should be interpreted descriptively, and the estimated heterogeneous associations cannot be causally attributed to any of the observed covariates.

## Data Availability

The datasets analyzed in this study are part of the Japan Gerontological Evaluation Study and are not publicly available because access is restricted by ethical and data use agreements. Deidentified data may be available from the Japan Gerontological Evaluation Study data management committee on reasonable request and subject to necessary approvals (contact: dataadmin.ml@jages.net). For analytic transparency, excerpts of the main analytic code for the targeted maximum likelihood estimation, generalized random forest, and supplementary analyses are provided in Multimedia Appendix 1.

## Appendix

Multimedia Appendix 1 Baseline covariates, descriptive statistics, sensitivity analyses, propensity score diagnostics, generalized random forest variable importance, communication modality–related variables, sample selection comparisons, supplementary figures, and main analytic code excerpts.

## Abbreviations

CATE: conditional average treatment effect
JAGES: Japan Gerontological Evaluation Study
TMLE: targeted maximum likelihood estimation
