# Supplementary material for: Heterogeneous Associations Between Frequent Virtual Communication and Loneliness Among Older Adults: Observational Analysis
Source: J Med Internet Res. 2026 Aug 7;28:e96187. doi: 10.2196/96187 (PMC13498908; doi:10.2196/96187)
Supplement: Supplementary file 1 [file jmir_v28i1e96187_app1.docx]

Table S1. List of Baseline Covariates.

| Variable Type | Measurement |
| --- | --- |
| Demographic | Age (years, continuous variable) |
|  | Gender (Men, Women) |
|  | Marital Status (Married, Widowed, Divorced, Single, Other) |
|  | Living Alone (Yes/No) |
|  | Employment Status (Working, Retired, Never worked) |
|  | Quit Job/Retired (Yes/No) |
|  | Started Living Alone (Yes/No) |
|  | Started Living with Children (Yes/No) |
|  | Started a New Job (Yes/No) |
|  | Lost Spouse (Yes/No) |
|  | Relocation Experience (Yes/No) |
| Socioeconomic | Educational Attainment (Less than 6 years, 6–9 years, 10–12 years, 13+ years, Other) |
|  | Annual household income (continuous variable) |
|  | Affluence at Age 15 (Upper, Middle, Lower) |
|  | Became Better Off Financially (Yes/No) |
|  | Became Worse Off Financially (Yes/No) |
| Health | Self-Rated Health (Bad, Not Good, Good, Very Good) |
|  | GDS15 (count variable, treated as continuous) |
|  | Ear Disease (Yes/No) |
|  | Eye Disease (Yes/No) |
|  | Number of Natural Teeth (ordinal variable: None, 1–4, 5–9, 10–19, 20+) |
|  | Suffered a Serious Illness (Yes/No) |
|  | Started Caring for Sick Family Members (Yes/No) |
|  | Self Maintenance^a^ (count variable, treated as continuous) |
|  | Intellectual Activity^b^ (count variable, treated as continuous) |
|  | Social Role^c^ (count variable, treated as continuous) |
| Psychosocial | Eating Alone (Yes/No) |
|  | Going Out Frequency (Once a week or more / A few times a month or less) |
|  | Parks/Foot Paths Suitable for Exercise (Relatively available / Relatively unavailable) |
|  | Civic Participation^d^ (count variable, treated as continuous) |
|  | Reciprocity^e^ (count variable, treated as continuous) |
|  | Social Cohesion^f^ (count variable, treated as continuous) |
|  | A Close Family Member or a Friend Passed Away (Yes/No) |
|  | Acquired New Friends (Yes/No) |
|  | Frequency of Meeting Friends (Four+ times weekly, Two to three times weekly, Weekly, Monthly (1–3 times), Annually (a few times), Rarely/Never.) |
|  | Number of Friends Met Last Month (None, 1–2, 3–5, 6–9, 10 or more) |
|  | Access to Fresh Foods (Relatively available / Relatively unavailable) |
| Behavioral | Smoking Status (Non-Smoker, Ex-Smoker, Smoker) |
|  | Alcohol Consumption (Drinker, Ex-Drinker, Non-Drinker) |
|  | Fish and Meat Consumption (Once a day or more / Less than once a day) |
|  | Vegetable/Fruit Consumption (Once a day or more / Less than once a day) |
|  | Internet Use 2018–2019 (A few times a month or less / A few times a week or more) |

Abbreviations: GDS: Geriatric Depression Scale; IADL: Instrumental Activity of Daily Living.

Self Maintenance^a^, Intellectual Activity^b^, and Social Role^c^ are the three components of IADL. Civic Participation^d^, Reciprocity^e^, and Social Cohesion^f^ represent the three key dimensions of Social Capital.

Table S2. Full descriptive statistics of imputed and original data on baseline covariates measured in 2019, frequent virtual communication assessed in 2022, and loneliness assessed in 2022 among older adults in Japan.

| Characteristics | Total (Imputed Data) | Total (Original Data) | **Frequent virtual communication users** | **Non-users/infrequent users** |
| --- | --- | --- | --- | --- |
|  | (n = 4807) | (n = 4807) | (n = 1616) | (n = 3191) |
| Loneliness in 2022^b^, mean (SD) | 4.2 (1.4) | 4.2 (1.4) | 3.9 (1.2) | 4.3 (1.5) |
| Age (years), mean (SD) | 73.7 (6) | 73.7 (6) | 71.2 (4.9) | 74.9 (6.1) |
| Gender, n (%) - Women | 2548 (53%) | 2548 (53%) | 926 (57%) | 1622 (51%) |
| Marital Status, n (%) |  |  |  |  |
| Married | 3607 (75%) | 3568 (74%) | 1303 (81%) | 2304 (72%) |
| Widowed | 853 (18%) | 810 (17%) | 221 (14%) | 632 (20%) |
| Divorced | 192 (4%) | 192 (4%) | 59 (4%) | 133 (4%) |
| Single | 128 (3%) | 128 (3%) | 29 (2%) | 99 (3%) |
| Other | 27 (1%) | 27 (1%) | 4 (0%) | 23 (1%) |
| Missing | 0 | 82 (2%) | 0 | 0 |
| Educational Attainment, n (%) |  |  |  |  |
| Less than 6 years | 26 (1%) | 26 (1%) | 2 (0%) | 24 (1%) |
| 6–9 years | 1296 (27%) | 1269 (26%) | 166 (10%) | 1130 (35%) |
| 10–12 years | 2096 (44%) | 2026 (42%) | 754 (47%) | 1342 (42%) |
| 13+ years | 1360 (28%) | 1359 (28%) | 686 (42%) | 674 (21%) |
| Other | 29 (1%) | 29 (1%) | 8 (0%) | 21 (1%) |
| Missing | 0 | 98 (2%) | 0 | 0 |
| Annual household income, mean (SD) (in 10000 yen) | 242.2 (146.2) | 247.2 (155.4) | 287.1 (153.0) | 219.5 (137.2) |
| Smoking Status, n (%) |  |  |  |  |
| Non-Smoker | 2924 (61%) | 2881 (60%) | 998 (62%) | 1926 (60%) |
| Ex-Smoker | 1431 (30%) | 1392 (29%) | 489 (30%) | 942 (30%) |
| Smoker | 452 (9%) | 452 (9%) | 129 (8%) | 323 (10%) |
| Missing | 0 | 82 (2%) | 0 | 0 |
| Alcohol Consumption, n (%) |  |  |  |  |
| Drinker | 1922 (40%) | 1921 (40%) | 710 (44%) | 1212 (38%) |
| Ex-Drinker | 631 (13%) | 458 (10%) | 180 (11%) | 451 (14%) |
| Non-Drinker | 2254 (47%) | 2228 (46%) | 726 (45%) | 1528 (48%) |
| Missing | 0 | 200 (4%) | 0 | 0 |
| Fish and Meat Consumption, n (%) - Once a day or more | 2546 (53%) | 2522 (52%) | 987 (61%) | 1559 (49%) |
| Vegetable/Fruit Consumption, n (%) - Once a day or more | 3887 (81%) | 3816 (79%) | 1388 (86%) | 2499 (78%) |
| Eating Alone, n (%) - Yes | 1040 (22%) | 1040 (22%) | 337 (21%) | 703 (22%) |
| Going Out Frequency, n (%) - Once a week or more | 4679 (97%) | 4627 (96%) | 1602 (99%) | 3077 (96%) |
| Parks/Foot Paths Suitable for Exercise, n (%) - Relatively available | 3555 (74%) | 3515 (73%) | 1300 (80%) | 2255 (71%) |
| Civic Participation^c^, mean (SD) | 1.1 (1.2) | 0.8 (1.1) | 1.4 (1.3) | 0.9 (1.2) |
| Reciprocity^d^, mean (SD) | 2.7 (0.6) | 2.7 (0.6) | 2.8 (0.5) | 2.7 (0.7) |
| Social Cohesion^e^, mean (SD) | 2.1 (1.1) | 2.1 (1.1) | 2.2 (1) | 2 (1.1) |
| Living Alone, n (%) |  |  |  |  |
| Yes | 571 (12%) | 566 (12%) | 161 (10%) | 410 (13%) |
| No | 4236 (88%) | 4139 (86%) | 1455 (90%) | 2781 (87%) |
| Missing | 0 | 102 (2%) | 0 | 0 |
| Employment Status, n (%) |  |  |  |  |
| Working | 1505 (31%) | 1479 (31%) | 614 (38%) | 891 (28%) |
| Retired | 3055 (64%) | 2702 (56%) | 952 (59%) | 2103 (66%) |
| Never worked | 247 (5%) | 247 (5%) | 50 (3%) | 197 (6%) |
| Missing | 0 | 379 (8%) | 0 | 0 |
| Quit Job/Retired, n (%) |  |  |  |  |
| Yes | 258 (5%) | 258 (5%) | 76 (5%) | 182 (6%) |
| No | 4549 (95%) | 4402 (92%) | 1540 (95%) | 3009 (94%) |
| Missing | 0 | 147 (3%) | 0 | 0 |
| Started Living Alone, n (%) |  |  |  |  |
| Yes | 100 (2%) | 100 (2%) | 18 (1%) | 82 (3%) |
| No | 4707 (98%) | 4560 (95%) | 1598 (99%) | 3109 (97%) |
| Missing | 0 | 147 (3%) | 0 | 0 |
| Started Living with Children, n (%) |  |  |  |  |
| Yes | 93 (2%) | 93 (2%) | 20 (1%) | 73 (2%) |
| No | 4714 (98%) | 4567 (95%) | 1596 (99%) | 3118 (98%) |
| Missing | 0 | 147 (3%) | 0 | 0 |
| Started a New Job, n (%) |  |  |  |  |
| Yes | 145 (3%) | 145 (3%) | 56 (3%) | 89 (3%) |
| No | 4662 (97%) | 4515 (94%) | 1560 (97%) | 3102 (97%) |
| Missing | 0 | 147 (3%) | 0 | 0 |
| Lost Spouse, n (%) |  |  |  |  |
| Yes | 134 (3%) | 134 (3%) | 23 (1%) | 111 (3%) |
| No | 4673 (97%) | 4526 (94%) | 1593 (99%) | 3080 (97%) |
| Missing | 0 | 147 (3%) | 0 | 0 |
| Became Better Off Financially, n (%) |  |  |  |  |
| Yes | 185 (4%) | 185 (4%) | 59 (4%) | 126 (4%) |
| No | 4622 (96%) | 4475 (93%) | 1557 (96%) | 3065 (96%) |
| Missing | 0 | 147 (3%) | 0 | 0 |
| Became Worse Off Financially, n (%) |  |  |  |  |
| Yes | 547 (11%) | 547 (11%) | 150 (9%) | 397 (12%) |
| No | 4260 (89%) | 4113 (86%) | 1466 (91%) | 2794 (88%) |
| Missing | 0 | 147 (3%) | 0 | 0 |
| A Close Family Member or a Friend Passed Away, n (%) |  |  |  |  |
| Yes | 1522 (32%) | 1516 (32%) | 519 (32%) | 1003 (31%) |
| No | 3285 (68%) | 3144 (65%) | 1097 (68%) | 2188 (69%) |
| Missing | 0 | 147 (3%) | 0 | 0 |
| Acquired New Friends, n (%) |  |  |  |  |
| Yes | 402 (8%) | 402 (8%) | 188 (12%) | 214 (7%) |
| No | 4405 (92%) | 4258 (89%) | 1428 (88%) | 2977 (93%) |
| Missing | 0 | 147 (3%) | 0 | 0 |
| Self-Rated Health, n (%) |  |  |  |  |
| Bad | 47 (1%) | 47 (1%) | 11 (1%) | 36 (1%) |
| Not Good | 536 (11%) | 528 (11%) | 117 (7%) | 419 (13%) |
| Good | 3496 (73%) | 3438 (72%) | 1192 (74%) | 2304 (72%) |
| Very Good | 728 (15%) | 728 (15%) | 296 (18%) | 432 (14%) |
| Missing | 0 | 66 (1%) | 0 | 0 |
| GDS15, mean (SD) | 2.9 (3) | 2.9 (3) | 2.2 (2.5) | 3.3 (3.1) |
| Ear Disease, n (%) |  |  |  |  |
| Yes | 275 (6%) | 275 (6%) | 63 (4%) | 212 (7%) |
| No | 4532 (94%) | 4320 (90%) | 1553 (96%) | 2979 (93%) |
| Missing | 0 | 212 (4%) | 0 | 0 |
| Eye Disease, n (%) |  |  |  |  |
| Yes | 834 (17%) | 833 (17%) | 261 (16%) | 573 (18%) |
| No | 3973 (83%) | 3762 (78%) | 1355 (84%) | 2618 (82%) |
| Missing | 0 | 212 (4%) | 0 | 0 |
| Number of Natural Teeth, n (%) |  |  |  |  |
| None | 355 (7%) | 355 (7%) | 52 (3%) | 303 (9%) |
| 1–4 | 266 (6%) | 266 (6%) | 45 (3%) | 221 (7%) |
| 5–9 | 494 (10%) | 440 (9%) | 94 (6%) | 400 (13%) |
| 10–19 | 1148 (24%) | 1057 (22%) | 320 (20%) | 828 (26%) |
| 20+ | 2544 (53%) | 2538 (53%) | 1105 (68%) | 1439 (45%) |
| Missing | 0 | 151 (3%) | 0 | 0 |
| Suffered a Serious Illness, n (%) |  |  |  |  |
| Yes | 267 (6%) | 267 (6%) | 73 (5%) | 194 (6%) |
| No | 4540 (94%) | 4393 (91%) | 1543 (95%) | 2997 (94%) |
| Missing | 0 | 147 (3%) | 0 | 0 |
| Started Caring for Sick Family Members, n (%) |  |  |  |  |
| Yes | 201 (4%) | 201 (4%) | 64 (4%) | 137 (4%) |
| No | 4606 (96%) | 4459 (93%) | 1552 (96%) | 3054 (96%) |
| Missing | 0 | 147 (3%) | 0 | 0 |
| Self Maintenance^f^, mean (SD) | 5.1 (0.5) | 5.1 (0.5) | 5.1 (0.3) | 5.1 (0.5) |
| Intellectual Activity^g^, mean (SD) | 4.4 (0.7) | 4.4 (0.7) | 4.3 (0.6) | 4.5 (0.8) |
| Social Role^h^, mean (SD) | 4.8 (1) | 4.8 (1.0) | 4.6 (0.9) | 4.9 (1.1) |
| Frequency of Meeting Friends, n (%) |  |  |  |  |
| Four+ times weekly | 725 (15%) | 725 (15%) | 305 (19%) | 420 (13%) |
| Two to three times weekly | 990 (21%) | 956 (20%) | 348 (22%) | 642 (20%) |
| Weekly | 677 (14%) | 632 (13%) | 245 (15%) | 432 (14%) |
| Monthly (1–3 times) | 1189 (25%) | 1158 (24%) | 417 (26%) | 772 (24%) |
| Annually (a few times) | 895 (19%) | 888 (18%) | 241 (15%) | 654 (20%) |
| Rarely/Never | 331 (7%) | 331 (7%) | 60 (4%) | 271 (8%) |
| Missing | 0 | 117 (2%) | 0 | 0 |
| Number of Friends Met Last Month, n (%) |  |  |  |  |
| None | 321 (7%) | 321 (7%) | 67 (4%) | 254 (8%) |
| 1–2 | 850 (18%) | 832 (17%) | 203 (13%) | 647 (20%) |
| 3–5 | 1313 (27%) | 1267 (26%) | 374 (23%) | 939 (29%) |
| 6–9 | 729 (15%) | 678 (14%) | 254 (16%) | 475 (15%) |
| 10+ | 1594 (33%) | 1589 (33%) | 718 (44%) | 876 (27%) |
| Missing | 0 | 120 (2%) | 0 | 0 |
| Relocation Experience, n (%) |  |  |  |  |
| Yes | 4676 (97%) | 4545 (95%) | 1568 (97%) | 3108 (97%) |
| No | 131 (3%) | 131 (3%) | 48 (3%) | 83 (3%) |
| Missing | 0 | 131 (3%) | 0 | 0 |
| Affluence at Age 15, n (%) |  |  |  |  |
| Upper | 559 (12%) | 559 (12%) | 243 (15%) | 316 (10%) |
| Middle | 2243 (47%) | 2158 (45%) | 805 (50%) | 1438 (45%) |
| Lower | 2005 (42%) | 1922 (40%) | 568 (35%) | 1437 (45%) |
| Missing | 0 | 168 (3%) | 0 | 0 |
| Access to Fresh Foods, n (%) - Relatively available | 3632 (76%) | 3570 (74%) | 1253 (78%) | 2379 (75%) |
| Internet Use 2018–2019, n (%) - A few times a month or less | 2635 (55%) | 2445 (51%) | 282 (17%) | 2353 (74%) |

Abbreviations: GDS: Geriatric Depression Scale; IADL: Instrumental Activity of Daily Living; SD: Standard Deviation.

Frequent virtual communicationa was defined using a frequency item for internet or email use and a communication-purpose item for communication with family, friends, or acquaintances at least two to three times per week during the preceding year. The communication-purpose item included examples such as email, LINE, Zoom, and video calls but did not separately identify each platform or modality. Non-users/infrequent users were defined as participants who did not meet this frequent virtual communication definition. Loneliness was assessed in 2022b using the UCLA Loneliness Scale (range: 3-9), with higher scores indicating greater loneliness. Civic Participationc, Reciprocityd, and Social Cohesione represent the three key dimensions of Social Capital. Self Maintenancef, Intellectual Activityg, and Social Roleh are the three components of IADL. Missing categories in the original data columns indicate pre-imputation missingness and are shown for descriptive purposes only. The final analytical models used the imputed analytic dataset; missingness was not treated as a separate category in the final analytical models.

Table S3. Full Baseline Covariates Measured in 2019 and Loneliness Assessed in 2022, Stratified by Quartiles of Estimated CATEs for the Association Between Frequent Virtual Communication^a^ and Loneliness

| Baseline Characteristics | Q1 | Q2 | Q3 | Q4 |
| --- | --- | --- | --- | --- |
| Estimated CATEs, mean (SD) | -0.21 (0.03) | -0.13 (0.02) | -0.07 (0.02) | -0.02 (0.03) |
| Loneliness in 2022^b^, mean (SD), range 3-9 | 4.5 (1.6) | 4.3 (1.5) | 4.1 (1.4) | 3.9 (1.3) |
| Age (years), mean (SD), range 65-94 | 73.2 (5.9) | 74.1 (6.1) | 74.0 (6.0) | 73.4 (5.8) |
| Gender, n (%) - Women | 51 (4%) | 376 (31%) | 960 (80%) | 1161 (97%) |
| Marital Status, n (%) |  |  |  |  |
| Married | 989 (82%) | 918 (76%) | 830 (69%) | 870 (72%) |
| Widowed | 93 (8%) | 196 (16%) | 284 (24%) | 280 (23%) |
| Divorced | 66 (5%) | 46 (4%) | 49 (4%) | 31 (3%) |
| Single | 48 (4%) | 33 (3%) | 26 (2%) | 21 (2%) |
| Other | 6 (0%) | 9 (1%) | 12 (1%) | 0 (0%) |
| Educational Attainment, n (%) |  |  |  |  |
| Less than 6 years | 12 (1%) | 9 (1%) | 2 (0%) | 3 (0%) |
| 6–9 years | 366 (30%) | 353 (29%) | 336 (28%) | 241 (20%) |
| 10–12 years | 301 (25%) | 524 (44%) | 559 (47%) | 712 (59%) |
| 13+ years | 515 (43%) | 308 (26%) | 298 (25%) | 239 (20%) |
| Other | 8 (1%) | 8 (1%) | 6 (0%) | 7 (1%) |
| Annual household income, mean (SD) (in 10000 yen), range 8.8-1200 | 240 (154) | 227 (143) | 233 (135) | 269 (150) |
| Smoking Status, n (%) |  |  |  |  |
| Non-Smoker | 81 (7%) | 600 (50%) | 1053 (88%) | 1190 (99%) |
| Ex-Smoker | 957 (80%) | 402 (33%) | 71 (6%) | 1 (0%) |
| Smoker | 164 (14%) | 200 (17%) | 77 (6%) | 11 (1%) |
| Alcohol Consumption, n (%) |  |  |  |  |
| Drinker | 266 (22%) | 423 (35%) | 737 (61%) | 828 (69%) |
| Ex-Drinker | 211 (18%) | 201 (17%) | 138 (11%) | 81 (7%) |
| Non-Drinker | 725 (60%) | 578 (48%) | 326 (27%) | 293 (24%) |
| Fish and Meat Consumption, n (%) - Once a day or more | 549 (46%) | 551 (46%) | 630 (52%) | 816 (68%) |
| Vegetable/Fruit Consumption, n (%) - Once a day or more | 870 (72%) | 910 (76%) | 1005 (84%) | 1102 (92%) |
| Eating Alone, n (%) - Yes | 248 (21%) | 255 (21%) | 303 (25%) | 234 (19%) |
| Going Out Frequency, n (%) - Once a week or more | 1167 (97%) | 1163 (97%) | 1172 (98%) | 1177 (98%) |
| Parks/Foot Paths Suitable for Exercise, n (%) - Relatively available | 892 (74%) | 875 (73%) | 854 (71%) | 934 (78%) |
| Civic Participation^c^, mean (SD), range 0-5 | 0.8 (1.1) | 1.0 (1.2) | 1.2 (1.3) | 1.3 (1.3) |
| Reciprocity^d^, mean (SD), range 0-3 | 2.5 (0.8) | 2.7 (0.6) | 2.7 (0.5) | 2.9 (0.3) |
| Social Cohesion^e^, mean (SD), range 0-3 | 2.0 (1.1) | 2.1 (1.1) | 2.1 (1.1) | 2.1 (1.0) |
| Living Alone, n (%) - Yes | 128 (11%) | 139 (12%) | 167 (14%) | 137 (11%) |
| Employment Status, n (%) |  |  |  |  |
| Working | 442 (37%) | 409 (34%) | 351 (29%) | 303 (25%) |
| Retired | 737 (61%) | 747 (62%) | 757 (63%) | 814 (68%) |
| Never worked | 23 (2%) | 46 (4%) | 93 (8%) | 85 (7%) |
| Quit Job/Retired, n (%) - Yes | 85 (7%) | 70 (6%) | 61 (5%) | 42 (3%) |
| Started Living Alone, n (%) - Yes | 25 (2%) | 20 (2%) | 32 (3%) | 23 (2%) |
| Started Living with Children, n (%) - Yes | 19 (2%) | 34 (3%) | 26 (2%) | 14 (1%) |
| Started a New Job, n (%) - Yes | 48 (4%) | 41 (3%) | 34 (3%) | 22 (2%) |
| Lost Spouse, n (%) - Yes | 22 (2%) | 28 (2%) | 41 (3%) | 43 (4%) |
| Became Better Off Financially, n (%) - Yes | 46 (4%) | 55 (5%) | 46 (4%) | 38 (3%) |
| Became Worse Off Financially, n (%) - Yes | 150 (12%) | 162 (13%) | 136 (11%) | 99 (8%) |
| A Close Family Member or a Friend Passed Away, n (%) - Yes | 244 (20%) | 405 (34%) | 367 (31%) | 506 (42%) |
| Acquired New Friends, n (%) - Yes | 81 (7%) | 114 (9%) | 88 (7%) | 119 (10%) |
| Self-Rated Health, n (%) |  |  |  |  |
| Bad | 19 (2%) | 9 (1%) | 11 (1%) | 8 (1%) |
| Not Good | 172 (14%) | 141 (12%) | 124 (10%) | 99 (8%) |
| Good | 849 (71%) | 831 (69%) | 873 (73%) | 943 (78%) |
| Very Good | 162 (13%) | 221 (18%) | 193 (16%) | 152 (13%) |
| GDS15, mean (SD), range 0-15 | 3.2 (3.3) | 3.1 (3.2) | 2.8 (2.8) | 2.7 (2.5) |
| Ear Disease, n (%) - Yes | 71 (6%) | 80 (7%) | 60 (5%) | 64 (5%) |
| Eye Disease, n (%) - Yes | 209 (17%) | 207 (17%) | 211 (18%) | 207 (17%) |
| Number of Natural Teeth, n (%) |  |  |  |  |
| None | 119 (10%) | 94 (8%) | 83 (7%) | 59 (5%) |
| 1–4 | 83 (7%) | 68 (6%) | 72 (6%) | 43 (4%) |
| 5–9 | 140 (12%) | 157 (13%) | 119 (10%) | 78 (6%) |
| 10–19 | 266 (22%) | 320 (27%) | 312 (26%) | 250 (21%) |
| 20+ | 594 (49%) | 563 (47%) | 615 (51%) | 772 (64%) |
| Suffered a Serious Illness, n (%) - Yes | 99 (8%) | 82 (7%) | 47 (4%) | 39 (3%) |
| Started Caring for Sick Family Members, n (%) - Yes | 44 (4%) | 41 (3%) | 52 (4%) | 64 (5%) |
| Self Maintenance^f^, mean (SD), range 5-10 | 5.16 (0.53) | 5.14 (0.56) | 5.07 (0.41) | 5.04 (0.31) |
| Intellectual Activity^g^, mean (SD), range 4-8 | 4.56 (0.80) | 4.53 (0.81) | 4.39 (0.69) | 4.30 (0.61) |
| Social Role^h^, mean (SD), range 4-8 | 5.12 (1.12) | 4.83 (1.03) | 4.64 (0.95) | 4.51 (0.80) |
| Frequency of Meeting Friends, n (%) |  |  |  |  |
| Four+ times weekly | 164 (14%) | 172 (14%) | 229 (19%) | 160 (13%) |
| Two to three times weekly | 164 (14%) | 232 (19%) | 284 (24%) | 310 (26%) |
| Weekly | 141 (12%) | 169 (14%) | 147 (12%) | 220 (18%) |
| Monthly (1–3 times) | 273 (23%) | 319 (27%) | 282 (23%) | 315 (26%) |
| Annually (a few times) | 327 (27%) | 215 (18%) | 196 (16%) | 157 (13%) |
| Rarely/Never | 133 (11%) | 95 (8%) | 63 (5%) | 40 (3%) |
| Number of Friends Met Last Month, n (%) |  |  |  |  |
| None | 144 (12%) | 90 (7%) | 55 (5%) | 32 (3%) |
| 1–2 | 267 (22%) | 209 (17%) | 191 (16%) | 183 (15%) |
| 3–5 | 322 (27%) | 382 (32%) | 346 (29%) | 263 (22%) |
| 6–9 | 145 (12%) | 168 (14%) | 194 (16%) | 222 (18%) |
| 10+ | 324 (27%) | 353 (29%) | 415 (35%) | 502 (42%) |
| Relocation Experience, n (%) - Yes | 1159 (96%) | 1169 (97%) | 1178 (98%) | 1170 (97%) |
| Affluence at Age 15, n (%) |  |  |  |  |
| Upper | 97 (8%) | 103 (9%) | 165 (14%) | 194 (16%) |
| Middle | 368 (31%) | 492 (41%) | 543 (45%) | 840 (70%) |
| Lower | 737 (61%) | 607 (50%) | 493 (41%) | 168 (14%) |
| Access to Fresh Foods, n (%) - Relatively available | 906 (75%) | 881 (73%) | 902 (75%) | 943 (78%) |
| Internet Use 2018–2019, n (%) - A few times a month or less | 636 (53%) | 688 (57%) | 701 (58%) | 610 (51%) |

Abbreviations: GDS: Geriatric Depression Scale; IADL: Instrumental Activity of Daily Living; SD: Standard Deviation.

Frequent virtual communicationa was defined using a frequency item for internet or email use and a communication-purpose item for communication with family, friends, or acquaintances at least two to three times per week during the preceding year. The communication-purpose item included examples such as email, LINE, Zoom, and video calls but did not separately identify each platform or modality. Non-users/infrequent users were defined as participants who did not meet this frequent virtual communication definition. Loneliness was assessed in 2022b using the UCLA Loneliness Scale (range: 3-9), with higher scores indicating greater loneliness. Civic Participationc, Reciprocityd, and Social Cohesione represent the three key dimensions of Social Capital. Self Maintenancef, Intellectual Activityg, and Social Roleh are the three components of IADL. CATE denotes the conditional average treatment effect estimand; estimated CATEs denote the individual-level forest predictions used for stratification. Because this study was an observational association analysis, the estimated CATEs are interpreted as exploratory heterogeneity in adjusted associations, not as definitive causal effects.

Table S4. Sensitivity analysis incorporating baseline loneliness in an alternative linked sample.

| **Model / sample** | **n** | **Exposed / reference n** | **Mean difference (95% CI)** | **P value** |
| --- | --- | --- | --- | --- |
| Main analytic sample | 4807 | 1616 / 3191 | -0.136 (-0.194 to -0.078) | <.001 |
| Alternative linked sample with baseline loneliness | 4682 | 1542 / 3140 | -0.117 (-0.173 to -0.061) | <.001 |

The sensitivity analysis in the alternative linked sample adjusted for baseline loneliness. The alternative linked sample was constructed by starting from 2019 respondents with baseline loneliness data, linking them to 2022 follow-up data, and applying the corresponding 2022 eligibility and exposure/outcome availability requirements. This yielded 4,682 participants. This analysis was supplementary because it used an alternative linked sample with baseline loneliness and the 2019 baseline general internet-use covariate was unavailable in this sample by design. Although assignment of the 2019 questionnaire modules was random, patterns of non-response to the baseline loneliness module might differ from those to the submodule used in the main analysis; therefore, distinct selection bias cannot be ruled out.

Table S5. Sensitivity analysis using a dichotomized loneliness outcome.

| **Outcome** | **n** | **Exposed / reference n** | **Log odds ratio** | **Odds ratio (95% CI)** | **P value** |
| --- | --- | --- | --- | --- | --- |
| Lonely: score 6 to 9; not lonely: score 3 to 5 | 4807 | 1616 / 3191 | -0.309 | 0.734 (0.597 to 0.904) | .004 |

The 3-item UCLA Loneliness Scale was dichotomized as lonely (scores 6 to 9) versus not lonely (scores 3 to 5) for this supplementary sensitivity analysis, following prior studies [31,32]. The odds ratio compares frequent virtual communication users with non-users/infrequent users.

Table S6. Propensity score overlap diagnostics.

**Panel A. Distribution of estimated propensity scores by exposure group.**

| **Metric** | **Non-users/infrequent users** | **Frequent virtual communication** |
| --- | --- | --- |
| n | 3191 | 1616 |
| Minimum | 0.000 | 0.009 |
| 1st percentile | 0.005 | 0.043 |
| 5th percentile | 0.013 | 0.113 |
| 25th percentile | 0.043 | 0.441 |
| Median | 0.107 | 0.656 |
| 75th percentile | 0.292 | 0.791 |
| 95th percentile | 0.696 | 0.904 |
| 99th percentile | 0.845 | 0.953 |
| Maximum | 0.965 | 1.000 |

**Panel B. Extreme propensity score and common support diagnostics.**

| **Metric** | **Non-users/infrequent users** | **Frequent virtual communication** |
| --- | --- | --- |
| Propensity score < 0.05 | 920 (28.83%) | 25 (1.55%) |
| Propensity score < 0.10 | 1552 (48.64%) | 70 (4.33%) |
| Propensity score > 0.90 | 8 (0.25%) | 90 (5.57%) |
| Propensity score > 0.95 | 2 (0.06%) | 22 (1.36%) |
| Outside common support | 85 (2.66%) | 9 (0.56%) |

The common support range was 0.009 to 0.965. Values are rounded to three decimals, and propensity scores were truncated to the interval [1e-6, 1 - 1e-6] before diagnostic summaries. These diagnostics were used to assess overlap between frequent virtual communication users and non-users/infrequent users. Limited overlap remains possible, particularly in the lower tail among non-users/infrequent users and in the upper tail among frequent virtual communication users.

Table S7. Variable importance from the generalized random forest, aggregated to baseline characteristics.

| **Rank** | **Baseline characteristic** | **Relative importance (%)** | **Model-matrix columns, n** |
| --- | --- | --- | --- |
| 1 | Frequency of meeting friends | 12.00 | 5 |
| 2 | Smoking status | 11.46 | 2 |
| 3 | Number of friends met in the past month | 9.10 | 4 |
| 4 | Sex | 8.51 | 2 |
| 5 | Educational attainment | 8.44 | 4 |
| 6 | Childhood socioeconomic status | 8.35 | 2 |
| 7 | Equivalized household income | 5.50 | 1 |
| 8 | Reciprocity | 4.08 | 1 |
| 9 | Depressive symptoms | 3.82 | 1 |
| 10 | Close family member or friend passed away | 3.76 | 1 |
| 11 | Number of natural teeth | 3.42 | 4 |
| 12 | Self-rated health | 2.09 | 3 |
| 13 | Social role | 2.01 | 1 |
| 14 | Social cohesion | 1.95 | 1 |
| 15 | Fish and meat consumption | 1.79 | 1 |
| 16 | Employment status | 1.79 | 2 |
| 17 | Civic participation | 1.78 | 1 |
| 18 | Age | 1.71 | 1 |
| 19 | Eye disease | 1.71 | 1 |
| 20 | Alcohol consumption | 1.57 | 2 |
| 21 | Vegetable/fruit consumption | 1.28 | 1 |
| 22 | Eating alone | 1.06 | 1 |
| 23 | Intellectual activity | 0.85 | 1 |
| 24 | Parks/foot paths suitable for exercise | 0.80 | 1 |
| 25 | Access to fresh foods | 0.44 | 1 |
| 26 | Internet use in 2018-2019 | 0.41 | 1 |
| 27 | Marital status | 0.22 | 4 |
| 28 | Living alone | 0.04 | 1 |
| 29 | Became worse off financially | 0.03 | 1 |
| 30 | Acquired new friends | 0.01 | 1 |
| 31 | Self maintenance | 0.00 | 1 |
| 32 | Ear disease | 0.00 | 1 |
| 33 | Started living alone | 0.00 | 1 |
| 34 | Started living with children | 0.00 | 1 |
| 35 | Started caring for sick family members | 0.00 | 1 |
| 36 | Suffered a serious illness | 0.00 | 1 |
| 37 | Became better off financially | 0.00 | 1 |
| 38 | Started a new job | 0.00 | 1 |
| 39 | Quit job/retired | 0.00 | 1 |
| 40 | Lost spouse | 0.00 | 1 |
| 41 | Going out frequency | 0.00 | 1 |
| 42 | Relocation experience | 0.00 | 1 |

Variable importance reflects how variables contributed to splitting in the generalized random forest. For categorical variables, dummy-coded model-matrix columns were aggregated back to the corresponding baseline characteristic. These values are algorithmic split-use diagnostics and do not imply that a variable is a conceptually important, independent, or causal source of heterogeneity. The heterogeneity analysis is exploratory.

Table S8. Communication-modality-related variables available in the 2022 questionnaire.

| **Relationship target** | **Mode** | **Frequent category** | **Frequent n (%)** | **Missing n (%)** |
| --- | --- | --- | --- | --- |
| Family or relatives living separately | In-person meeting | Almost every day or two to three times per week | 1074 (22.34%) | 210 (4.37%) |
| Family or relatives living separately | Video call (face visible) | Almost every day or two to three times per week | 253 (5.26%) | 463 (9.63%) |
| Family or relatives living separately | Letter, telephone, or email (face not visible) | Almost every day or two to three times per week | 1440 (29.96%) | 261 (5.43%) |
| Friends or acquaintances | In-person meeting | Almost every day or two to three times per week | 1543 (32.1%) | 210 (4.37%) |
| Friends or acquaintances | Video call (face visible) | Almost every day or two to three times per week | 166 (3.45%) | 411 (8.55%) |
| Friends or acquaintances | Letter, telephone, or email (face not visible) | Almost every day or two to three times per week | 1242 (25.84%) | 251 (5.22%) |

These modality-related variables are not identical to the primary exposure definition. The primary exposure item did not separately identify email, LINE, Zoom, or video calls. These variables are reported only to describe available communication-mode information and to support the limitation that the primary exposure combined heterogeneous communication modes. Frequent category refers to almost every day or two to three times per week; percentages use the analytic sample denominator of 4807 unless otherwise indicated. The frequent-use counts for video calls were relatively small, supporting the decision not to stratify the primary exposure by modality.

Table S9. Baseline characteristics of the 2019 survey respondents and the final analytic sample.

| **Characteristics** | **2019 survey respondents (n = 24769)** | **Analytic sample (n = 4807)** | **Abs. SMD** |
| --- | --- | --- | --- |
| Age (years), mean (SD), range | 74.8 (6.4); 65 to 95 | 73.7 (6.0); 65 to 94 | 0.173 |
| Gender, n (%) |  |  |  |
| Men | 11,663 (47.1%) | 2,259 (47.0%) | 0.002 |
| Women | 13,106 (52.9%) | 2,548 (53.0%) | 0.002 |
| Marital Status, n (%) |  |  |  |
| Married | 17,571 (70.9%) | 3,568 (74.2%) | 0.074 |
| Widowed | 4,602 (18.6%) | 810 (16.9%) | 0.045 |
| Divorced | 1,108 (4.5%) | 192 (4.0%) | 0.024 |
| Single | 755 (3.0%) | 128 (2.7%) | 0.023 |
| Other | 193 (0.8%) | 27 (0.6%) | 0.027 |
| Missing | 540 (2.2%) | 82 (1.7%) | 0.034 |
| Living Alone, n (%) |  |  |  |
| No | 20,573 (83.1%) | 4,139 (86.1%) | 0.084 |
| Yes | 3,607 (14.6%) | 566 (11.8%) | 0.083 |
| Missing | 589 (2.4%) | 102 (2.1%) | 0.017 |
| Employment Status, n (%) |  |  |  |
| Working | 6,783 (27.4%) | 1,479 (30.8%) | 0.075 |
| Retired | 14,227 (57.4%) | 2,702 (56.2%) | 0.025 |
| Never worked | 1,480 (6.0%) | 247 (5.1%) | 0.037 |
| Missing | 2,279 (9.2%) | 379 (7.9%) | 0.047 |
| Quit Job/Retired, n (%) |  |  |  |
| No | 22,501 (90.8%) | 4,402 (91.6%) | 0.026 |
| Yes | 1,308 (5.3%) | 258 (5.4%) | 0.004 |
| Missing | 960 (3.9%) | 147 (3.1%) | 0.045 |
| Started Living Alone, n (%) |  |  |  |
| No | 23,171 (93.5%) | 4,560 (94.9%) | 0.056 |
| Yes | 638 (2.6%) | 100 (2.1%) | 0.033 |
| Missing | 960 (3.9%) | 147 (3.1%) | 0.045 |
| Started Living with Children, n (%) |  |  |  |
| No | 23,312 (94.1%) | 4,567 (95.0%) | 0.039 |
| Yes | 497 (2.0%) | 93 (1.9%) | 0.005 |
| Missing | 960 (3.9%) | 147 (3.1%) | 0.045 |
| Started a New Job, n (%) |  |  |  |
| No | 23,038 (93.0%) | 4,515 (93.9%) | 0.037 |
| Yes | 771 (3.1%) | 145 (3.0%) | 0.006 |
| Missing | 960 (3.9%) | 147 (3.1%) | 0.045 |
| Lost Spouse, n (%) |  |  |  |
| No | 23,106 (93.3%) | 4,526 (94.2%) | 0.036 |
| Yes | 703 (2.8%) | 134 (2.8%) | 0.003 |
| Missing | 960 (3.9%) | 147 (3.1%) | 0.045 |
| Relocation Experience, n (%) |  |  |  |
| No | 829 (3.3%) | 131 (2.7%) | 0.036 |
| Yes | 23,128 (93.4%) | 4,545 (94.5%) | 0.049 |
| Missing | 812 (3.3%) | 131 (2.7%) | 0.032 |
| Educational Attainment, n (%) |  |  |  |
| Less than 6 years | 186 (0.8%) | 26 (0.5%) | 0.026 |
| 6-9 years | 6,223 (25.1%) | 1,269 (26.4%) | 0.029 |
| 10-12 years | 10,313 (41.6%) | 2,026 (42.1%) | 0.010 |
| 13+ years | 7,192 (29.0%) | 1,359 (28.3%) | 0.017 |
| Other | 202 (0.8%) | 29 (0.6%) | 0.025 |
| Missing | 653 (2.6%) | 98 (2.0%) | 0.040 |
| Annual household income, mean (SD), range (in 10000 yen) | 244.1 (158.5); 8.3 to 1200 | 247.2 (155.4); 8.8 to 1200 | 0.020 |
| Affluence at Age 15, n (%) |  |  |  |
| Upper | 3,323 (13.4%) | 559 (11.6%) | 0.054 |
| Middle | 10,759 (43.4%) | 2,158 (44.9%) | 0.029 |
| Lower | 9,536 (38.5%) | 1,922 (40.0%) | 0.030 |
| Missing | 1,151 (4.6%) | 168 (3.5%) | 0.058 |
| Became Better Off Financially, n (%) |  |  |  |
| No | 22,855 (92.3%) | 4,475 (93.1%) | 0.032 |
| Yes | 954 (3.9%) | 185 (3.8%) | 0.000 |
| Missing | 960 (3.9%) | 147 (3.1%) | 0.045 |
| Became Worse Off Financially, n (%) |  |  |  |
| No | 21,134 (85.3%) | 4,113 (85.6%) | 0.007 |
| Yes | 2,675 (10.8%) | 547 (11.4%) | 0.018 |
| Missing | 960 (3.9%) | 147 (3.1%) | 0.045 |
| Self-Rated Health, n (%) |  |  |  |
| Bad | 473 (1.9%) | 47 (1.0%) | 0.078 |
| Not Good | 3,015 (12.2%) | 528 (11.0%) | 0.037 |
| Good | 17,377 (70.2%) | 3,438 (71.5%) | 0.030 |
| Very Good | 3,464 (14.0%) | 728 (15.1%) | 0.033 |
| Missing | 440 (1.8%) | 66 (1.4%) | 0.032 |
| GDS15, mean (SD), range | 3.0 (3.1); 0 to 15 | 2.9 (3.0); 0 to 15 | 0.047 |
| Ear Disease, n (%) |  |  |  |
| No | 22,062 (89.1%) | 4,320 (89.9%) | 0.026 |
| Yes | 1,495 (6.0%) | 275 (5.7%) | 0.013 |
| Missing | 1,212 (4.9%) | 212 (4.4%) | 0.023 |
| Eye Disease, n (%) |  |  |  |
| No | 19,141 (77.3%) | 3,762 (78.3%) | 0.024 |
| Yes | 4,416 (17.8%) | 833 (17.3%) | 0.013 |
| Missing | 1,212 (4.9%) | 212 (4.4%) | 0.023 |
| Number of Natural Teeth, n (%) |  |  |  |
| None | 2,003 (8.1%) | 355 (7.4%) | 0.026 |
| 1-4 | 1,413 (5.7%) | 266 (5.5%) | 0.007 |
| 5-9 | 2,288 (9.2%) | 440 (9.2%) | 0.003 |
| 10-19 | 5,126 (20.7%) | 1,057 (22.0%) | 0.032 |
| 20+ | 13,022 (52.6%) | 2,538 (52.8%) | 0.004 |
| Missing | 917 (3.7%) | 151 (3.1%) | 0.031 |
| Suffered a Serious Illness, n (%) |  |  |  |
| No | 21,995 (88.8%) | 4,393 (91.4%) | 0.087 |
| Yes | 1,814 (7.3%) | 267 (5.6%) | 0.072 |
| Missing | 960 (3.9%) | 147 (3.1%) | 0.045 |
| Started Caring for Sick Family Members, n (%) |  |  |  |
| No | 22,807 (92.1%) | 4,459 (92.8%) | 0.026 |
| Yes | 1,002 (4.0%) | 201 (4.2%) | 0.007 |
| Missing | 960 (3.9%) | 147 (3.1%) | 0.045 |
| Self Maintenance, mean (SD), range | 5.2 (0.6); 5 to 10 | 5.1 (0.5); 5 to 10 | 0.101 |
| Intellectual Activity, mean (SD), range | 4.5 (0.8); 4 to 8 | 4.4 (0.7); 4 to 8 | 0.083 |
| Social Role, mean (SD), range | 4.9 (1.1); 4 to 8 | 4.8 (1.0); 4 to 8 | 0.130 |
| Eating Alone, n (%) |  |  |  |
| No | 18,740 (75.7%) | 3,767 (78.4%) | 0.064 |
| Yes | 6,029 (24.3%) | 1,040 (21.6%) | 0.064 |
| Going Out Frequency, n (%) |  |  |  |
| Once a week or more | 23,479 (94.8%) | 4,627 (96.3%) | 0.071 |
| A few times a month or less | 1,003 (4.0%) | 128 (2.7%) | 0.077 |
| Missing | 287 (1.2%) | 52 (1.1%) | 0.007 |
| Parks/Foot Paths Suitable for Exercise, n (%) |  |  |  |
| Relatively available | 18,720 (75.6%) | 3,515 (73.1%) | 0.056 |
| Relatively unavailable | 5,555 (22.4%) | 1,226 (25.5%) | 0.072 |
| Missing | 494 (2.0%) | 66 (1.4%) | 0.048 |
| Civic Participation, mean (SD), range | 0.8 (1.1); 0 to 5 | 0.8 (1.1); 0 to 5 | 0.021 |
| Reciprocity, mean (SD), range | 2.7 (0.6); 0 to 3 | 2.7 (0.6); 0 to 3 | 0.064 |
| Social Cohesion, mean (SD), range | 2.0 (1.1); 0 to 3 | 2.1 (1.1); 0 to 3 | 0.046 |
| A Close Family Member or a Friend Passed Away, n (%) |  |  |  |
| No | 16,249 (65.6%) | 3,144 (65.4%) | 0.004 |
| Yes | 7,560 (30.5%) | 1,516 (31.5%) | 0.022 |
| Missing | 960 (3.9%) | 147 (3.1%) | 0.045 |
| Acquired New Friends, n (%) |  |  |  |
| No | 21,756 (87.8%) | 4,258 (88.6%) | 0.023 |
| Yes | 2,053 (8.3%) | 402 (8.4%) | 0.003 |
| Missing | 960 (3.9%) | 147 (3.1%) | 0.045 |
| Frequency of Meeting Friends, n (%) |  |  |  |
| Four+ times weekly | 3,517 (14.2%) | 725 (15.1%) | 0.025 |
| Two or three times weekly | 4,682 (18.9%) | 956 (19.9%) | 0.025 |
| Weekly | 3,110 (12.6%) | 632 (13.1%) | 0.018 |
| Monthly (1-3 times) | 5,805 (23.4%) | 1,158 (24.1%) | 0.015 |
| Annually (a few times) | 4,771 (19.3%) | 888 (18.5%) | 0.020 |
| Rarely/Never | 2,129 (8.6%) | 331 (6.9%) | 0.064 |
| Missing | 755 (3.0%) | 117 (2.4%) | 0.038 |
| Number of Friends Met Last Month, n (%) |  |  |  |
| None | 2,230 (9.0%) | 321 (6.7%) | 0.087 |
| 1-2 | 4,760 (19.2%) | 832 (17.3%) | 0.049 |
| 3-5 | 6,094 (24.6%) | 1,267 (26.4%) | 0.040 |
| 6-9 | 3,260 (13.2%) | 678 (14.1%) | 0.027 |
| 10+ | 7,634 (30.8%) | 1,589 (33.1%) | 0.048 |
| Missing | 791 (3.2%) | 120 (2.5%) | 0.042 |
| Access to Fresh Foods, n (%) |  |  |  |
| Relatively available | 19,417 (78.4%) | 3,570 (74.3%) | 0.097 |
| Relatively unavailable | 4,860 (19.6%) | 1,161 (24.2%) | 0.110 |
| Missing | 492 (2.0%) | 76 (1.6%) | 0.031 |
| Smoking Status, n (%) |  |  |  |
| Non-Smoker | 14,439 (58.3%) | 2,881 (59.9%) | 0.033 |
| Ex-Smoker | 7,317 (29.5%) | 1,392 (29.0%) | 0.013 |
| Smoker | 2,537 (10.2%) | 452 (9.4%) | 0.028 |
| Missing | 476 (1.9%) | 82 (1.7%) | 0.016 |
| Alcohol Consumption, n (%) |  |  |  |
| Drinker | 9,767 (39.4%) | 1,921 (40.0%) | 0.011 |
| Ex-Drinker | 2,582 (10.4%) | 458 (9.5%) | 0.030 |
| Non-Drinker | 11,445 (46.2%) | 2,228 (46.3%) | 0.003 |
| Missing | 975 (3.9%) | 200 (4.2%) | 0.011 |
| Fish and Meat Consumption, n (%) |  |  |  |
| Once a day or more | 12,699 (51.3%) | 2,522 (52.5%) | 0.024 |
| A few times a week or less | 11,508 (46.5%) | 2,174 (45.2%) | 0.025 |
| Missing | 562 (2.3%) | 111 (2.3%) | 0.003 |
| Vegetable/Fruit Consumption, n (%) |  |  |  |
| Once a day or more | 19,233 (77.6%) | 3,816 (79.4%) | 0.042 |
| A few times a week or less | 5,006 (20.2%) | 908 (18.9%) | 0.033 |
| Missing | 530 (2.1%) | 83 (1.7%) | 0.030 |
| Internet Use 2018-2019, n (%) |  |  |  |
| A few times a month or less | 12,667 (51.1%) | 2,445 (50.9%) | 0.006 |
| A few times a week or more | 10,682 (43.1%) | 2,120 (44.1%) | 0.020 |
| Missing | 1,420 (5.7%) | 242 (5.0%) | 0.031 |

Abbreviations: GDS: Geriatric Depression Scale; IADL: Instrumental Activity of Daily Living; SD: Standard Deviation; SMD: standardized mean difference.

Values are mean (SD), range for continuous variables and n (%) for categorical variables. The 2019 survey respondents were those available in the distributed individual-level data. The pre-response initial enrollment group was not included because baseline covariates were not available for nonrespondents. Both the 2019 survey respondent sample and the final analytic sample were summarized using original, non-imputed data. Missing categories indicate pre-imputation missingness. Continuous-variable summaries were calculated among participants with observed values. Absolute SMDs are shown. The main analytical models used the imputed analytic dataset.

Figure S1. Directed acyclic graph illustrating the assumed temporal and reporting structure for baseline loneliness, baseline general internet use, retrospective exposure reporting, and loneliness in 2022.

The diagram summarizes the assumed relationships considered in the sensitivity analysis and limitations discussion. Baseline loneliness was not available in the main analytic sample. Baseline general internet use may partly capture prior digital engagement, while current loneliness may affect retrospective reporting of prior virtual communication. The diagram is a conceptual aid for interpretation and does not remove the possibility of residual confounding or recall bias.

Figure S2. Propensity score overlap for frequent virtual communication and non-users/infrequent users among older adults in the analytic sample.

The dotted vertical lines indicate the common support range. The comparison group is labeled as non-users/infrequent users because it includes true non-users and participants who did not meet the frequent virtual communication definition.

**Supplementary Code Excerpts**

The following excerpts summarize the R workflow used for the analyses. File paths, personal identifiers, and display-only commands were omitted. The excerpts preserve the random seeds, learner library, tree number, covariate set, exposure definition, and the 3-item UCLA Loneliness Scale specification.

**Code Excerpt S1. Analysis variables and covariate groups.**

demographic_factors2 <- c("age_meibo19", "sex_meibo19", "mari5st19", "hous2no19")
ses_factors2 <- c("educ5_19", "empl3pt19", "income4", "ssl15_5_19")
health_factors2 <- c(
 "srh_4_19", "GDS15", "IADL_SM", "IADL_IA", "IADL_SR",
 "teeth5_19", "dgns2vi19", "dgns2he19"
)
psychosocial_factors2 <- c(
 "meet6fr19", "num5fr19", "mov3no19", "Reciprocity",
 "Social_Cohesion", "Civic_Participation", "evnt2gj19", "evnt2rs19",
 "evnt2cc19", "evnt2al19", "evnt2er19", "evnt2fd19", "evnt2sd19",
 "evnt2cd19", "evnt2fr19", "evnt2di19", "evnt2cr19"
)
behavioral_factors2 <- c(
 "smoking3", "drinking3", "fq7prt19", "fq7veg19", "gout7fq19",
 "ml2al19", "env5prk19", "env5grc19", "inet4use19"
)

covariates2 <- c(
 demographic_factors2, ses_factors2, health_factors2,
 psychosocial_factors2, behavioral_factors2
)
treatment_var <- "internet_use"
outcome_var <- "loneliness"
SL.library <- c("SL.glm", "SL.xgboost", "SL.nnet")

**Code Excerpt S2. Exposure and 3-item UCLA loneliness score.**

\# Each UCLA item was scored 1-3; the total score ranged from 3 to 9.
recode_ucla3_item <- function(x) {
 pmin(pmax(round(x), 1), 3)
}

completed_data <- completed_data %>%
 mutate(
 inet4use22_group = ifelse(inet4use22 %in% c(3, 4), 1, 0),
 inet2cm22_group = ifelse(inet2cm22 == 1, 1, 0),
 internet_use = ifelse(inet4use22_group == 1 & inet2cm22_group == 1, 1, 0),
 soc3lac22_new = recode_ucla3_item(soc3lac22),
 soc3lon22_new = recode_ucla3_item(soc3lon22),
 soc3iso22_new = recode_ucla3_item(soc3iso22),
 loneliness = soc3lac22_new + soc3lon22_new + soc3iso22_new
 )

**Code Excerpt S3. Missing-data imputation and categorical-value restoration.**

imputed_data <- run_missForest_cached(
 data = extracted_data,
 output_dir = output_dir,
 cache_prefix = "missForest_2019_2022",
 seed = 123L,
 ntree = 50L,
 maxiter = 5L,
 parallelize = "forests",
 force_recompute = force_recompute_missForest
)
completed_data <- as.data.frame(imputed_data$ximp)

\# categories is a named list of allowable values for each categorical variable.
discretize_to_categories <- function(value, categories) {
 sapply(value, function(x) categories[which.min(abs(categories - x))])
}
for (var in names(categories)) {
 if (var %in% names(completed_data)) {
 completed_data[[var]] <- discretize_to_categories(completed_data[[var]], categories[[var]])
 }
}

**Code Excerpt S4. TMLE with SuperLearner for the adjusted mean difference.**

extract.estimates.ltmle <- function(fit) {
 output.TMLE <- summary(fit)$effect.measures
 summary.TMLE <- rbind(
 as.data.frame(output.TMLE$ATE),
 as.data.frame(output.TMLE$RR),
 as.data.frame(output.TMLE$treatment),
 as.data.frame(output.TMLE$control)
 )
 summary.TMLE %>% dplyr::filter(long.name == "Additive Treatment Effect")
}

ate.ltmle.loneliness <- extracted_data3 %>%
 dplyr::select(dplyr::all_of(c(covariates2, treatment_var, outcome_var))) %>%
 ltmle(
 Anodes = treatment_var,
 Ynodes = outcome_var,
 abar = list(1, 0),
 SL.library = SL.library,
 Yrange = range(.[[outcome_var]], na.rm = TRUE)
 )

ATE.estimates.loneliness <- ate.ltmle.loneliness %>%
 extract.estimates.ltmle() %>%
 mutate(outcome = "loneliness") %>%
 rename(CI.low = CI.2.5., CI.up = CI.97.5., p.value = pvalue) %>%
 dplyr::select(outcome, estimate, CI.low, CI.up, p.value)

**Code Excerpt S5. Generalized random forest and calibration.**

set.seed(12345)
num.folds <- 10
folds_vec <- sort(seq(nrow(extracted_data3)) %% num.folds) + 1
\# folds_vec was used to construct fold-specific estimated-CATE quartiles for descriptive summaries.
\# Forest predictions were obtained from the generalized random forest honest/out-of-bag prediction mechanism.
X_matrix <- model.matrix(~ . - 1, data = extracted_data3[, covariates2, with = FALSE])

forest.loneliness <- grf::causal_forest(
 X = X_matrix,
 Y = extracted_data3[[outcome_var]],
 W = extracted_data3[[treatment_var]],
 num.trees = 10000,
 tune.parameters = "all",
 clusters = folds_vec
)

preds.loneliness <- predict(forest.loneliness, estimate.variance = FALSE)$predictions
calibration_results <- grf::test_calibration(forest.loneliness) %>%
 broom::tidy() %>%
 mutate(outcome = outcome_var) %>%
 dplyr::select(outcome, term, estimate, std.error, p.value)

**Code Excerpt S6. Fold-specific quartiles of estimated CATEs.**

\# Estimated CATEs are forest predictions used to rank participants; they are not treated as the true CATEs themselves.
\# build_foldwise_quartiles_added() assigns quartiles within folds and handles ties.
extracted_data3$CATE_quartile <- build_foldwise_quartiles_added(
 preds = preds.loneliness,
 folds = folds_vec,
 labels = paste0("Q", 1:4)
)

ate.ranking.result <- ATE.ranking(
 forest = forest.loneliness,
 outcome = outcome_var,
 data = as.data.frame(extracted_data3),
 covariates = covariates2,
 treatment_var = treatment_var,
 folds = folds_vec,
 num.rankings = 4
)

**Code Excerpt S7. Supplementary sensitivity analyses and diagnostics.**

\# Dichotomized loneliness outcome sensitivity analysis.

model_data <- as.data.frame(extracted_data3[, c(covariates2, treatment_var, outcome_var), with = FALSE])

\# Binary loneliness was defined as a 3-item UCLA Loneliness Scale score of 6 or higher for the supplementary sensitivity analysis.

model_data$lonely_binary <- ifelse(

model_data[[outcome_var]] >= 6 & model_data[[outcome_var]] <= 9, 1,

ifelse(model_data[[outcome_var]] >= 3 & model_data[[outcome_var]] <= 5, 0, NA_real_)

)
binary_fit <- stats::glm(
 stats::as.formula(paste("lonely_binary ~", paste(c(treatment_var, covariates2), collapse = " + "))),
 data = model_data,
 family = stats::binomial()
)

\# Baseline loneliness sensitivity analysis used the alternative linked sample with baseline loneliness.
h_data$baseline_loneliness <-
 recode_ucla3_item(h_data$soc3lac19) +
 recode_ucla3_item(h_data$soc3lon19) +
 recode_ucla3_item(h_data$soc3iso19)

\# This sensitivity covariate set replaced baseline general internet use with baseline loneliness.
covariates2_h <- c(
 setdiff(covariates2, "inet4use19"),
 "baseline_loneliness"
)

h_fit <- ltmle(
 data = as.data.frame(h_data[, c(covariates2_h, treatment_var, outcome_var), with = FALSE]),
 Anodes = treatment_var,
 Ynodes = outcome_var,
 abar = list(1, 0),
 SL.library = SL.library,
 Yrange = range(h_data[[outcome_var]], na.rm = TRUE)
)

\# Propensity score overlap diagnostics.
propensity_formula <- stats::as.formula(paste(treatment_var, "~", paste(covariates2, collapse = " + ")))
ps_fit <- stats::glm(propensity_formula, data = model_data, family = stats::binomial())
propensity_score <- pmin(pmax(as.numeric(stats::predict(ps_fit, type = "response")), 1e-6), 1 - 1e-6)

\# Variable importance from the generalized random forest, aggregated to source characteristics.
variable_importance_bundle <- summarize_variable_importance_by_source_added(
 forest_object = forest.loneliness,
 matrix_columns = colnames(X_matrix),
 source_vars = covariates2
)
